# Effect of drainage ratio during strawberry cultivation:The volatilomics-based shelf-life indicators for strawberry fruit

**DOI:** 10.3389/fpls.2023.1124827

**Published:** 2023-03-21

**Authors:** Jwa Yeong Cho, Da Hye Ryu, Muhammad Hamayun, Soo Hyun Park, Ho-Youn Kim

**Affiliations:** ^1^ Smart Farm Research Center, Korea Institute of Science and Technology (KIST), Gangneung, Republic of Korea; ^2^ Division of Bio-Medical Science and Technology, KIST School, University of Science and Technology (UST), Daejeon, Republic of Korea; ^3^ Department of Botany, Abdul Wali Khan University, Mardan, Pakistan

**Keywords:** drainage ratio, shelf-life response factors, shelf-life, fruit quality, anthocyanin, volatile organic compound

## Abstract

The metabolome of strawberries at harvest determines their storage capacity. Therefore, dynamics of volatile production during storage of strawberry cultivated under diverse drainage ratios, T1 (12.0%), T2 (25.3%), T3 (36.4%), and T4 (56.5%), were evaluated. Among the various non-target VOCs analysis, there were some groups including aldehydes, esters, and furans occupied over 5% with exhibiting high coefficient of determination (*R^2^*) following the days after storage (DAS). Aldehydes content decreased over the storage period, while the esters (methyl butanoate, methyl hexanoate, ethyl hexanoate, and benzyl acetate) and furanones (furaneol and mesifuran) were increased as representing aroma compounds in strawberry ripening. Even on the same day, it was investigated that the release of VOCs linked to fruit decay was delayed in the groups (T1 and T2) that were given relatively little water compared to T3 and T4. The hexanal and ethyl hexanoate as an over-ripened signal showed a rapid increase from 4 DAS to 5 DAS in T3 and T4, respectively, while T1 and T2 showed significant increase from 5 DAS to 6 DAS. Relatively slower over-ripening tendency of T1 and T2 was supported by changes of firmness, total soluble solid content, anthocyanin content, and antioxidant activity during storage. T1 and T2 showed higher antioxidant activity at the harvest time and lower anthocyanin accumulation than T3 and T4. The present study elucidated that the preharvest drainage changes during cultivation was involved in fruit quality during strawberry storage. Besides, volatilomics analysis depicted that T2 as an optimal ratio, could delay the occurrence of stress and senescence, and guaranteed the strawberry yield. In conclusion, this study provided evidence that the practical application of drainage ratios could improve horticultural product quality even with low water use and VOCs might be considered an early indicator for strawberry fruit shelf-life.

## 1 Introduction

Aroma is main characteristics associated with the strawberry quality along with sweetness, hardness, and color. Particularly, aroma is key factors attracting customers, and these are dependent on certain volatile organic compounds (VOCs) (Fan et al., 2021). Although strawberry contain a small amount of VOCs, these flavor-related dominant compounds have been studied for their importance in consumer acceptance. So far, more than 360 volatile compounds have been identified in strawberries, but only a few VOCs, including C6 aldehydes, terpenes, esters, and furanones, contribute a lot to their aroma (Ulrich et al., 2018; Yan et al., 2018). In detail, linalool and nerolidol are main terpenes and esters constitute 25-90% of the total amount of VOCs in strawberry. Particularly, furanones such as furaneol and mesifuran provide the sweet aromas, whereas aldehydes including hexanal and (E)-2-hexanal contribute to the fresh aromas (Yan et al., 2018).

During postharvest storage, an increase in abscisic acid in fruit induces fruit senescence, anthocyanin accumulation (Setha, 2012; Qu et al., 2018), and VOCs emissions (Jiang and Joyce, 2003; Gagne et al., 2011). Especially, fruit ripening results in fluctuation in the concentrations of specific VOCs such as aldehydes, alcohols, and esters (Porta and Rocha-Sosa, 2002; Lv et al., 2014; Xu et al., 2017). As strawberry are vulnerable to mechanical injury, water loss, and fungi-caused decay, it has been recognized as one of the most perishable and delicate fruits (Giampieri et al., 2012; Hashmi et al., 2013; de Souza et al., 2014; Neri et al., 2015; Park et al., 2020). Its quality is fluctuated and can be determined by color, texture, and chemical composition, e.g. anthocyanin, total phenolic compounds, antioxidant activity as well as VOCs (Gunness et al., 2009). There have been a lot of efforts to maintain the marketability of fresh strawberries during the postharvest.

Common practices used to improve strawberry shelf-life are based on the postharvest treatment such as CA (controlled atmosphere) storage (Popa et al., 2019), coating with thymol or NaCl (Amal et al., 2010), and treating 1-MCP, ClO_2_ (Yang et al., 2018), oligo-chitosan application (Yan et al., 2012), and phytohormone application (Gimënez et al., 2014). As the result of these approaches, the composition of strawberry VOCs can be sensitively changed in a postharvest treatment dependent manner (Lu et al., 2018). To avoid these deleterious effects, the development of new strategies to preserve strawberry aroma is strongly suggested and required

Among the various techniques applied during cultivation, one of the major factors influencing strawberry quality is the controlled irrigation system, particularly the drainage system. It is possible to monitor daily nutrition uptake in plants and predict water uptake according to environmental conditions (Borin et al., 2001). Optimal drainage ratio is important for strawberry fruit yield and quality as it facilitates the accumulation of excess water and salts and provides high proportions of air in the rhizosphere (Gurovich and Oyarce, 2015). In addition, the controlled drainage with the raised-bed system in a greenhouse minimizes herbicide use and increases fruit quality and production (Yuan et al., 2004). Drainage systems represent simple and effective ways for suitable water supply to strawberry plants during cultivation (Lozano et al., 2016). However, the optimal drainage ratio improving fruit yield, quality, aroma, and shelf-life, it is still unknown for strawberry fruits.

In this study, we collected the VOCs of strawberries under four different drainage ratios during the storage to observe the change of VOCs distribution and figure out the volatile markers that can be used as an early shelf-life indicator. To support this, firmness, TSS, anthocyanin, and antioxidant were evaluated in this experiment. Therefore, it is possible to confirm the effect of preharvest treatment on postharvest shelf-life. Furthermore, this field trial will provide a reference for the practical application of drainage ratio and VOCs in agriculture.

## 2 Materials and methods

### 2.1 Description of the experimental design and environments

**Figure 1:**
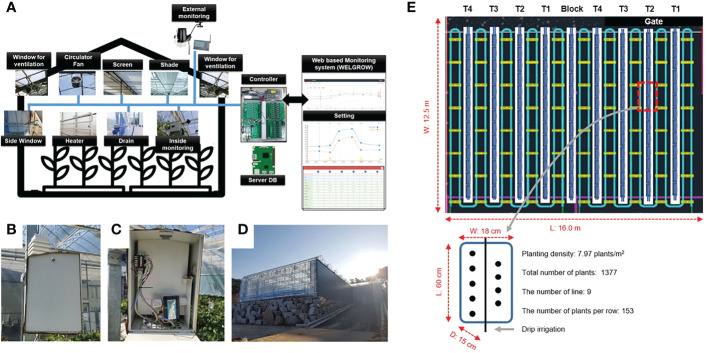
Overview diagram of the WELGROW system which monitors and controls the greenhouse climate **(A)**. Temperature, humidity, and CO_2_ sensors for monitoring the environment inside the greenhouse **(B, C)**, External view of the multi-span Venlo-type greenhouse **(D)**. Experimental design of strawberry growth inside the greenhouse **(E)**.

An experimental greenhouse at Korea Institute of Science and Technology (KIST), Gangneung, which is a multi-span Venlo-type structure, was used for the experiment (**Figure 1**). **Figure 1A** shows that room climate was control by various sensors on Web based monitoring system. Temperature, relative humidity, and CO_2_ concentration of the inside climate were measured by the sensor module (SH-VT250, Soha tech, Korea). It was installed at the center of the greenhouse with height of 1 meter (**Figures 1B, C**) and the specifications of the sensors are shown in **Table 1**. The crop used in the experiment was “Seolhayng” strawberries were used as the crops for the experiment. The size of cultivation room is 16 m in length and 12.5 m in width (**Figure 1**). The experiment was conducted 7 weeks after the strawberry seedlings were on Semp. 10^th^ 2019. A total number of 1377 plants were used in this experiment, and it was cultivated with a planting density of 7.97 plants/m^2^. Strawberries were grown in a greenhouse under a 24/9°C, day/night temperature cycle based on the mixed soil (80% pearlite with cocopeat; 10% zeolite; and 10% peat moss). The crops were provided with nutrients with major elements (4.27 mM Ca, 11.50 mM NO3, 4.52 mM K, 1.50 mM H2PO4, 1.50 mM SO4, and 1.50 mM Mg) and minor elements (29.98 mM Fe, 20.00 Mn, 6.98 mM Zn, 1.00 mM Cu, 11.98 mM B, and 0.50 mM Mo). Drainage ratios were considered as the treatments (T) and four drainage ratios including 12.0 ± 2.0% (T1), 25.3 ± 6.6% (T2), 36.4 ± 4.4% (T3), and 56.5 ± 4.5% (T4) were applied from fruit set using the automatic nutrient solution supply (Aqua-2ZT, Shinhan A-Tec, Changwon, Republic of Korea) determined the feeding time and E.C. by the accumulated solar radiation (**Figure 1**). The ratio of the amount of solution supplied for each experiment is shown in the following **Table 1**. The drainage ratios were calculated as


=(A-B) × 100%,

**Table 1:** Solution supplied for each treatment by the accumulated solar radiation.

Treatment	Flow per minute (L/min)	Drainage ratio against the supply				
		October 30^th^, 2019	November 5^th^, 2019	November 19^th^, 2019	December 20^th^, 2019	January 30^th^, 2020
T1	1	2362.5L (11.0%)	2808L (10.9%)	1620L (10.0%)	2060L (14.9%)	1520L (13.2%)
T2	2	7762.5L (18.2%)	11241L (22.1%)	7357.5L (23.1%)	9780L (35.3%)	6600L (27.8%)
T3	3	22473L (30.8%)	32625L (37.6%)	18360L (33.7%)	20450L (42.6%)	15020L (37.4%)
T4	4	52551L (53.3%)	60030L (50.6%)	43560L (59.3%)	41000L (61.6%)	28000L (57.8%)

where the A represent the total water supply and B means drainage after irrigation supply.

The harvested fruits were stored at room temperature for 6 days until there is decay on the strawberry surface. Each group was further divided into sub-groups according to date (0-6 days after storage, DAS) and then packed in polyethylene terephthalate (PET) containers.

### 2.2 Estimation of total soluble solids and fruit firmness

Total soluble solids (TSS) were measured using a refractometer (SCM-1000, HM Digital, Korea) and expressed in percentage (%). Fruit firmness was analyzed using a fruit hardness tester (Lutron FR5105, Taiwan) and expressed as N.

### 2.3 Estimation of antioxidants and anthocyanin

To analyze fruit antioxidant and anthocyanin concentrations, 2 g of frozen fruit was extracted with 4 mL of 80% methanol containing 0.1% HCl (hydrogen chloride) in a sonicator at 40°C for 1 h. Fruit extracts were filtered with a filter membrane (0.22 μm PVDF syringe filter, Maidstone, UK) and concentrated using a nitric evaporator (Allsheng MD 200, Hangzhou, China). Concentrated samples were dissolved in DMSO.

#### 2.3.1 DPPH free-radical scavenging activity

Antioxidant activity of samples on the day of harvesting was measured using the DPPH de-colorization method (Singh and Rajini, 2004). DPPH reagent was dissolved in ethanol to obtain an absorbance 1.00 (± 0.02) at 517 mm. Diverse concentrations of samples (10 μL) and DPPH solvent (190 μL) were reacted at 25°C for 30 min in the dark. After the reaction, absorbance was measured at 517 nm and antioxidant activity (%) was calculated as


$$=\left(1-\frac{Abs_{sample}-\;Abs_{blank}}{Abs_{control}}\right)\;\times \;100\%,$$

where the Abs_control_ indicates the reaction of ethanol and DMSO, Abs_sample_ means the mixture of sample and DPPH reagent, and Abs_blank_ explain the action of sample and ethanol. The derived activity was expressed as RC_50_ values, representing the concentration at which 50% of DPPH radicals were scavenged.

#### 2.3.2 Anthocyanin analysis

The anthocyanin contents of strawberry fruits were analyzed according to an established protocol (Lee et al., 2005). The anthocyanin standards for analytical purity, cyanidin-3-glucoside chloride (Sigma-Aldrich, St. Louis, USA), and pelargonidin-3-glucoside chloride (Cayman Chemical, Ann Arbor, Michigan, USA), were used. High-performance liquid chromatography (HPLC; Agilent 1200 series, Santa Clara, CA, USA) consisting of an auto-sampler, and binary pump system coupled with a diode array detector (DAD) was employed for qualitative and quantitative analysis. A reverse-phase C18 column (Supersil ODS-II; 250 mm × 4.6 mm, 5.0 μm particle sizes) setting temperature of 40°C and mobile phase consisted of 0.1% formic acid water as A solvent and 0.1% formic acid acetonitrile as B solvent with gradient elution mode (0-2 min from 80 to 70% B; 2-8 min from 70-55% B) were used for the sample analysis. The sequentially eluted components were detected at 520 nm through DAD, and these components were qualified through spiky test with standard compounds. Calibration curves, which were accepted based on the linearity (*R^2^*) over 0.999 in the ranges were constructed with five concentrations of each compound.

### 2.4 Headspace-solid phase microextraction (HS-SPME) analysis

The aromatic compounds of strawberry fruits were analyzed according to an established protocol (Cämara et al., 2006). The analysis was carried out using a gas chromatography (GC) system coupled to a time-of-flight mass spectrometer (TOF-MS) (LECO Pegasus GC HRT, Leco Corp., St. Joseph, MI, USA). Frozen sample (100 mg) was mixed with 2 mL of 30% NaCl (saturated solution) and 2 μL of 2 mg mL^-1^ 3-pentanol (used as the internal standard). An SPME holder equipped with a 50/30 μm fiber (DVB/CAR/PDMS, model 57348-U, Supelco Inc., PA, USA) was used for sampling. Before use, the fiber was conditioned at 250°C for 5 min. The aromatic compounds were extracted by exposure to SPME fiber maintained at 70°C and agitated at 500 rpm for 20 min. After the agitation, GC analysis was performed through capillary column (Rtx-5MS column, 30 m length, 0.25 mm diameter, 0.25 μm thickness, 5% diphenyl, 95% dimethyl polysiloxane, Restek, PA, USA) under split mode (30: 1). The front inlet temperature was set at 240°C and the transfer line temperature was maintained at 255°C. Mass range was from 36 to 450. The ion source temperature was kept at 250°C with ionization voltage of 70 eV and the helium was used as carrier gas at a flow rate of 1 ml min^-1^; oven temperature program was initial at 60°C held for 1 min and then 100°C at the rate of 8°C min^-1^ and then 220°C at the rate of 15°C min^-1^ and then programmed to 245°C at the rate of 30°C min^-1^ and finally held for 3 min. The relative quantity of these compounds was calculated based on the concentration of internal standard. Retention indices (RI) were determined from the retention times of a series of n-alkanes (C_6_–C_19_) with linear interpolation. VOCs of samples were identified on the basis of RIs and comparatively analyzed with the recorded mass spectra of each compound using mass spectrum library search (NIST).

### 2.5 Statistical analysis

The data were subjected to Analysis of Variance and Duncan’s new multiple range tests, performed in JMP *V*10.0.0 (SAS Institute, Cary, NC). In the illustrations, different capital letters between different storage duration treatments indicate significant differences and different lowercase letters indicate significant differences among treatments. Each treatment had three replicates and the experiments were carried out twice.

## 3 Results

### 3.1 Estimation of fruit production, firmness, and TSS

**Figure 2:**
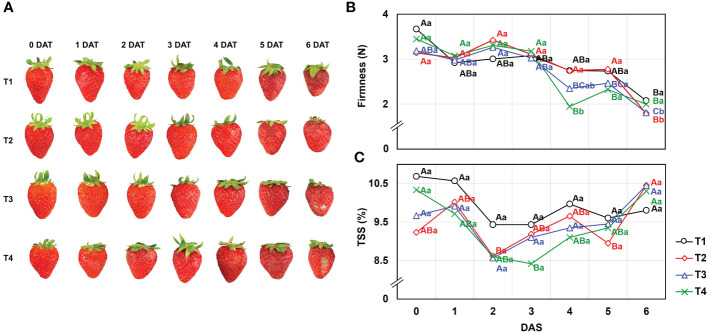
Effect of different drainage ratio treatments on **(A)** appearance, **(B)** firmness **(N)**, and **(C)** TSS (%) of strawberry fruit during day after storage (DAS) at room temperature (25°C). Means of values indicated with similar capital letters are not significantly different according to Tukey’s HSD test, p< 0.05. We used both capital and lowercase letters to illustrate the effect of storage duration and drainage ratio on fruit quality. Different capital letters **(A–C)** indicate significant differences under same drainage ratio treatments and lowercase letters (a, b) indicate significant differences among treatments in the course of storage. Drainage ratios represented as following: T1 (12.0%), T2 (25.3%), T3 (36.4%), and T4 (56.5%). DAS, days after storage; TSS, total soluble solids.

Higher fruit yield was obtained in T2 (25.2 ± 1.3 g) and T3 (25.5 ± 0.9 g), while lower production was observed under the T1 (22.6 ± 0.7 g) and T4 (19.3 ± 0.7 g) conditions (**Table 2**). In addition to fruit production, the effects of different drainage ratios on the quality of strawberry fruits were investigated by firmness and TSS during the days after storage (DAS) (**Figure 2**). For the firmness on the harvest day (0DAS), there was non-significant difference (*P*< 0.05) following the treatments as follows: T1 (3.67 ± 0.32 N), T2 (3.45 ± 0.31 N), T3 (3.18 ± 0.85 N), and T4 (3.14 ± 0.43 N). Additionally, the TSS on the harvest day (0 DAS) also explained no difference (*P*< 0.05) following the treatments; T1 (10.7 ± 1.12%), T2 (9.2 ± 0.62%), T3 (9.7 ± 1.15%), and T4 (10.3 ± 0.79%), respectively. TSS showed little effect of storage period as no significant difference (*P*< 0.05) was confirmed between 0 DAS and 6 DAS. But as presented in **Figure 2B**, the firmness gradually decreased as the storage period elapsed, and a significant difference (*P*< 0.05) was confirmed in all treatment groups at 6 DAS. However, T2 (1.80 ± 0.06 N) and T3 (1.81 ± 0.07 N) showed significantly lower firmness than T1 (2.08 ± 0.12 N) and T4 (2.00 ± 0.06 N) at 6 DAS.

**Table 2:** Strawberry fruit yield under different drainage ratio (*n* = 20) treatments.

Treatment	Yield (g per each fruit)
T1	22.6 ± 0.7^a^
T2	25.2 ± 1.3^a^
T3	25.5 ± 0.9^a^
T4	19.3 ± 0.7^b^

Data express as mean ± standard error. Drainage ratios represented as following: T1 (12.0%), T2 (25.3%), T3 (36.4%), and T4 (56.5%).

### 3.2 Fruit anthocyanins content and antioxidant activity

**Figure 3:**
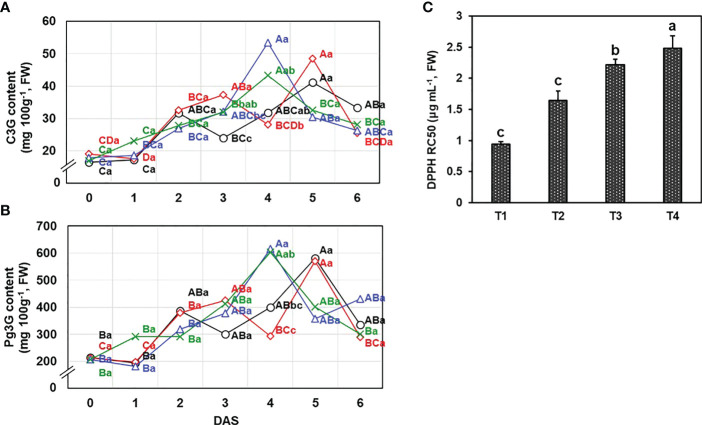
Effects of different drainage ratio treatments on anthocyanin compounds including **(A)** cyanidin-3-*O*-glucoside (C3G) and **(B)** pelargonidin-3-*O*-glucoside (Pg3G) was revealed by quantification using HPLC and **(C)** the antioxidant activity was evaluated by DPPH radical scavenging activity and result was expressed by RC_50_ value (mg mL^-1^). Different capital letters **(A–C)** indicate significant differences under same drainage ratio treatments and lowercase letters (a, b) indicate significant differences among treatments in the course of storage. Drainage ratios represented as following: T1 (12.0%), T2 (25.3%), T3 (36.4%), and T4 (56.5%). DAS, days after storage.

Pelargonidin-3-*O*-glucoside (Pg3G) and cyanidin-3-*O*-glucoside (C3G) are common anthocyanin compound in all strawberry varieties. “Seolhyang” is reported to mainly contain Pg3G consistent with the distribution of Pg3G (50-90% of the total anthocyanins) in previous studies (Dzhanfezova et al., 2020). Instrumental analysis using HPLC-DAD allowed the separation of anthocyanin peaks detected at 520 nm, showing retention time of 3.294 min of C3G and 4.114 min of Pg3G. Diverse concentrations were applied to draw calibration curves (C3G: 5-50 μg mL^-1^; Pg3G: 50-500 μg mL^-1^) and linear calibration curves of C3G (Y = 24.057X + 4.3388, *R^2^* = 0.9997) and Pg3G (Y = 2.1287X + 4.3673, *R^2^* = 0.9995) were obtained. Following the conditions and DAS, Pg3G (1803.1-6161.0 mg kg^-1^, FW) and C3G (164.0-534.0 mg kg^-1^, FW) were varied (**Figure 3**). The anthocyanins content increased up to the maximum content of each treatment after harvest and then declined later on. Two drainage conditions (T3 and T4) had the maximum anthocyanin contents at 4 DAS; however, T1 and T2 recorded the highest anthocyanins content at 5 DAS.

On the day of harvest, there was no difference (*P*< 0.05) in anthocyanins content and the total phenolic content (TPC) (**Supplementary Figure 1**) according to the drainage conditions, but a significant difference in antioxidant activity was observed. The antioxidant activity of fruit has been reported to indicate storability in early harvest stages (Oz et al., 2016) and can be decided by the secondary metabolites (Park et al., 2020). There were significant differences in DPPH free-radical scavenging activity, expressed as RC_50_ value, on harvest time (*P*< 0.05) (**Figure 3C**). The group with the lowest drainage ratio exhibited the highest antioxidant activity (T1 = 0.94 ± 0.04 mg mL^-1^ > T2 = 1.64 ± 0.15 mg mL^-1^ > T3 = 2.22 ± 0.09 mg mL^-1^ > T4 = 2.48 ± 0.20 mg mL^-1^).

### 3.3 Profiling of fruit volatile organic compounds (VOCs)

It is well known that the variability of VOCs according to the environment and characteristic properties changing during the storage period make VOCs the quality evaluation index (Fan et al., 2021; Öz and Kafkas, 2022). Because the VOCs of strawberry fruit can be used as an indicator of over-ripening during storage, 47 VOCs from the strawberry fruits collected were identified (**Table 3**), and these VOCs were associated with monoterpene alcohols (4), sesquiterpene alcohols (3), aldehydes (7), ketone (1), esters (25), lactone (1), monoterpene (1), sesquiterpenes (3), and furans (2).

**Table 3:** Profiling of volatile organic compounds (VOCs) in strawberry fruits during the storage (mg 100g^-1^).

	Tentative identification	R.I	0 DAS				1 DAS				2 DAS				3 DAS				4 DAS				5 DAS				6 DAS			
	*Aldehydes*																													
**1**	Hexanal	804	32.86	±	13.76	b	17.74	±	4.05	bc	20.62	±	10.25	bc	16.69	±	7.53	bc	10.67	±	2.78	c	28.48	±	18.97	ab	117.82	±	32.33	a
**2**	(E)-2-Hexenal	854	186.11	±	67.49	a	171.14	±	15.39	ab	165.78	±	31.62	ab	137.28	±	50.95	bc	117.81	±	28.38	c	99.50	±	18.51	c	100.19	±	21.59	c
**3**	Heptanal	901	13.41	±	23.69	a	0.43	±	0.12	b	0.43	±	0.09	b	0.37	±	0.12	b	0.19	±	0.09	b	0.36	±	0.09	b	0.72	±	0.14	b
**4**	(E,E)-2,4-Hexadienal	908	0.93	±	0.47	a	0.47	±	0.14	b	0.46	±	0.33	b	0.19	±	0.13	bc	0.13	±	0.06	c	0.02	±	0.01	c	0.03	±	0.01	c
**5**	Benzaldehyde	963	0.15	±	0.13	c	0.27	±	0.11	bc	0.14	±	0.06	c	0.38	±	0.30	bc	0.38	±	0.13	bc	0.40	±	0.22	b	1.02	±	0.31	a
**6**	Octanal	1005	0.62	±	0.15	b	0.56	±	0.17	b	0.51	±	0.15	b	0.42	±	0.16	b	5.95	±	6.67	a	1.35	±	1.13	b	3.36	±	2.30	ab
**7**	Decanal	1205	0.68	±	0.18	b	0.84	±	0.30	b	1.10	±	0.29	b	1.07	±	0.39	b	1.32	±	0.43	ab	1.36	±	0.23	ab	2.64	±	3.06	a
	***Sum of aldehydes***		234.76	±	102.56	a	191.44	±	14.14	ab	189.03	±	41.74	ab	156.40	±	55.93	b	136.45	±	29.37	b	131.48	±	16.91	b	225.77	±	35.97	a
	*Esters*																													
**8**	Methyl butanoate	723	2.75	±	1.78	e	8.69	±	2.56	d	12.45	±	2.31	cd	19.12	±	5.23	b	16.45	±	4.59	bc	24.33	±	6.09	a	16.36	±	4.56	bc
**9**	Methyl 2-methylbutyrate	785	0.38	±	0.30	c	0.39	±	0.18	c	0.60	±	0.18	c	0.98	±	0.50	c	0.92	±	0.26	c	2.90	±	1.33	b	5.31	±	2.12	a
**10**	Isopropyl butyrate	846	0.03	±	0.02	b	0.31	±	0.09	a	0.42	±	0.13	a	0.44	±	0.18	a	0.31	±	0.12	a	0.43	±	0.07	a	0.28	±	0.23	a
**11**	Methyl hexanoate	926	16.29	±	3.16	d	25.32	±	7.43	bc	27.54	±	7.42	b	23.36	±	7.24	bcd	19.63	±	4.01	cd	30.42	±	5.43	b	43.88	±	5.17	a
**12**	Ethyl hexanoate	998	10.71	±	9.49	b	5.99	±	4.49	b	2.14	±	1.15	b	8.76	±	7.39	b	7.99	±	6.22	b	51.46	±	52.74	b	359.43	±	114.56	a
**13**	Hexyl acetate	1011	5.74	±	2.83	b	5.01	±	2.16	b	3.40	±	1.15	b	4.48	±	1.13	b	6.25	±	4.29	b	39.37	±	23.06	a	29.26	±	29.38	a
**14**	2-Hexen-1-yl acetate	1019	14.24	±	7.02	ab	15.21	±	7.00	ab	11.20	±	4.55	ab	13.40	±	7.87	ab	14.15	±	6.07	ab	20.67	±	11.94	a	8.59	±	8.48	b
**15**	Methyl octanoate	1026	0.59	±	0.25	c	1.07	±	0.27	c	1.04	±	0.40	c	1.28	±	0.44	c	1.57	±	0.46	c	4.21	±	0.77	b	7.34	±	1.98	a
**16**	Benzyl acetate	1164	2.29	±	1.04	d	10.34	±	3.23	d	23.28	±	7.37	c	48.05	±	13.07	ab	50.51	±	13.74	a	38.95	±	10.03	b	23.93	±	5.79	c
**17**	Ethyl benzoate	1172	0.37	±	0.12	c	1.02	±	0.37	c	0.96	±	0.33	c	3.15	±	2.09	c	1.43	±	0.25	c	8.74	±	4.34	b	21.64	±	5.52	a
**18**	2-(2-Butoxyethoxy)ethanol	1192	5.48	±	1.39	e	6.90	±	1.08	de	7.95	±	1.31	cde	12.24	±	4.20	bcd	15.11	±	1.64	b	21.21	±	9.83	a	13.04	±	5.42	bc
**19**	Ethyl octanoate	1194	0.68	±	0.74	c	0.25	±	0.26	c	0.10	±	0.04	c	0.28	±	0.16	c	0.80	±	1.07	c	14.77	±	12.89	b	69.51	±	15.57	a
**20**	Methyl salicylate	1195	4.38	±	1.04	c	4.07	±	2.44	c	6.63	±	2.00	bc	6.61	±	3.26	bc	5.67	±	2.85	bc	10.85	±	1.38	a	9.28	±	6.98	ab
**21**	Octyl acetate	1211	0.60	±	0.29	b	1.44	±	0.53	b	1.63	±	0.44	b	3.21	±	0.94	b	2.79	±	0.68	b	5.33	±	3.77	b	35.56	±	20.55	a
**22**	2-Nonanol, acetate	1236	0.20	±	0.13	d	0.82	±	0.32	d	0.89	±	0.30	cd	1.53	±	0.70	c	1.55	±	0.15	c	2.27	±	0.28	b	3.56	±	1.16	a
**23**	Isopentyl hexanoate	1245	0.24	±	0.34	c	0.20	±	0.10	c	0.25	±	0.14	c	0.73	±	0.42	c	1.12	±	0.24	c	2.86	±	1.28	b	4.42	±	2.16	a
**24**	Octanoic acid, 3-hydroxy-, methyl ester	1253	1.13	±	1.23	d	2.07	±	0.99	d	1.63	±	1.14	d	8.13	±	6.56	cd	15.61	±	7.81	c	32.94	±	11.24	b	63.21	±	16.31	a
**25**	Triacetin	1346	0.36	±	0.07	d	0.52	±	0.08	cd	0.59	±	0.14	c	0.91	±	0.24	b	0.93	±	0.12	b	1.65	±	0.25	a	1.71	±	0.18	a
**26**	2-(2-Butoxyethoxy)ethyl acetate	1366	5.26	±	1.09	c	7.21	±	0.94	bc	9.20	±	1.98	b	11.26	±	2.15	a	11.23	±	1.65	a	12.51	±	1.78	a	12.10	±	1.34	a
**27**	Octyl 2-methylbutyrate	1442	0.25	±	0.16	c	0.78	±	0.23	c	1.07	±	0.49	c	3.49	±	1.66	b	2.93	±	0.64	b	5.65	±	0.98	a	6.18	±	1.17	a
**28**	Acetic acid, cinnamyl ester	1449	0.10	±	0.07	e	2.57	±	0.86	e	21.89	±	1.22	de	8.15	±	3.65	c	7.05	±	1.83	cd	13.38	±	3.95	b	22.52	±	7.12	a
**29**	Octyl hexanoate	1575	0.41	±	0.60	c	1.08	±	1.12	c	1.75	±	2.36	c	10.56	±	7.48	c	11.36	±	4.60	c	43.26	±	14.89	b	70.40	±	19.32	a
	***Sum of esters***		72.50	±	13.15	d	101.27	±	15.44	cd	117.62	±	22.80	cd	190.12	±	48.08	c	195.35	±	20.38	c	388.16	±	107.98	b	827.50	±	172.38	a
	*Monoterpene*																													
**30**	β-Ocimene	1049	0.20	±	0.12	b	0.17	±	0.03	bc	0.20	±	0.06	b	0.33	±	0.10	a	0.38	±	0.11	a	0.35	±	0.07	a	0.07	±	0.08	c
	***Sum of monoterpenes***		0.20	±	0.12	b	0.17	±	0.03	bc	0.20	±	0.06	b	0.33	±	0.10	a	0.38	±	0.11	a	0.35	±	0.07	a	0.07	±	0.08	c
	*Sesquiterpenes*																													
**31**	(Z)-β-Famesene	1442	2.03	±	1.51	e	6.75	±	1.47	d	7.22	±	2.88	d	16.42	±	4.83	bc	14.67	±	3.44	c	20.38	±	3.74	ab	21.09	±	5.03	a
**32**	(E)-β-Farnesene	1459	0.86	±	0.70	d	2.81	±	0.81	cd	3.12	±	1.45	c	7.33	±	2.35	ab	5.82	±	1.56	b	8.75	±	2.10	a	8.92	±	1.35	a
**33**	(Z,E)-α-Farnesene	1479	0.51	±	0.35	d	1.79	±	0.41	c	1.93	±	0.88	c	4.16	±	1.50	ab	3.36	±	0.73	b	5.06	±	1.08	a	5.23	±	1.00	a
	***Sum of sesquiterpenes***		3.40	±	2.50	e	11.35	±	2.50	d	12.28	±	5.12	d	27.91	±	8.62	bc	23.85	±	5.19	c	34.19	±	6.83	ab	35.24	±	7.10	a
	*Acids*																													
**34**	2-Ethylhexanoic acid	1125	0.77	±	0.23	de	0.71	±	0.07	e	0.81	±	0.15	de	0.97	±	0.20	cd	1.04	±	0.13	bc	1.24	±	0.20	ab	1.29	±	0.22	a
**35**	Nonanoic acid	1276	8.72	±	1.92	b	10.83	±	1.66	ab	10.59	±	2.35	ab	11.21	±	2.62	ab	13.88	±	4.52	a	11.80	±	1.98	ab	14.14	±	8.32	a
**36**	Hydrocinnamic acid	1350	0.00	±	0.00	b	0.00	±	0.00	b	0.00	±	0.00	b	0.06	±	0.03	b	0.07	±	0.02	b	0.18	±	0.15	a	0.19	±	0.09	a
	***Sum of acids***		9.49	±	2.04	b	11.54	±	1.69	ab	11.40	±	2.43	ab	12.25	±	2.78	ab	14.98	±	4.59	a	13.23	±	2.14	ab	15.62	±	8.30	a
	*Lactone*																													
**37**	γ-Dodecalactone	1472	3.83	±	2.39	d	16.57	±	3.89	bc	12.53	±	5.49	cd	26.32	±	10.59	ab	17.29	±	6.97	bc	28.92	±	13.64	a	29.60	±	7.73	a
	***Sum of lactones***		3.83	±	2.39	d	16.57	±	3.89	bc	12.53	±	5.49	cd	26.32	±	10.59	ab	17.29	±	6.97	bc	28.92	±	13.64	a	29.60	±	7.73	a
	*Ketone*																													
**38**	2-Heptanone	891	1.31	±	0.61	e	3.03	±	0.71	cde	2.75	±	1.03	de	5.38	±	2.76	bc	4.46	±	0.98	cd	6.99	±	1.73	ab	8.75	±	3.71	a
	***Sum of ketones***		1.31	±	0.61	e	3.03	±	0.71	cde	2.75	±	1.03	de	5.38	±	2.76	bc	4.46	±	0.98	cd	6.99	±	1.73	ab	8.75	±	3.71	a
	*Furans*																													
**39**	Mesifuran	1067	14.65	±	8.41	f	36.88	±	5.78	ef	47.86	±	16.01	e	116.20	±	31.44	d	154.66	±	22.82	c	208.87	±	27.76	b	315.77	±	28.24	a
**40**	Furaneol	1081	1.12	±	0.72	c	1.62	±	1.09	c	1.80	±	1.97	c	5.17	±	4.79	c	8.78	±	7.68	c	44.26	±	7.79	b	60.10	±	11.29	a
	***Sum of furans***		15.77	±	8.90	f	38.49	±	5.79	ef	49.67	±	17.74	e	121.37	±	31.24	d	163.44	±	25.10	c	253.13	±	31.93	b	375.87	±	29.06	a
	*Monoterpene alcohols*																													
**41**	(E)-Linalool oxide	1086	2.83	±	0.75	b	4.13	±	1.04	b	3.22	±	1.16	b	5.21	±	1.60	b	5.03	±	0.90	b	4.94	±	3.54	b	9.72	±	3.34	a
**42**	Linalool	1099	24.41	±	2.43	d	57.28	±	6.40	c	65.56	±	13.96	bc	91.59	±	24.42	a	87.25	±	20.55	a	77.80	±	11.85	ab	79.34	±	4.99	ab
**43**	L-α-Terpineol	1198	1.53	±	0.35	d	3.62	±	0.95	cd	5.00	±	1.28	c	9.19	±	2.62	b	14.11	±	4.63	a	12.12	±	3.24	ab	11.70	±	2.03	ab
**44**	Nerol	1248	0.26	±	0.10	e	0.53	±	0.14	de	0.87	±	0.32	cd	1.18	±	0.39	bc	2.04	±	0.75	a	1.52	±	0.19	b	1.44	±	0.48	b
	***Sum of monoterpene alcohols***		29.03	±	2.65	d	65.57	±	6.63	c	74.65	±	16.20	bc	107.17	±	28.16	a	108.44	±	25.67	a	96.37	±	16.86	ab	102.19	±	5.87	a
	*Sesquiterpenes*																													
**45**	Nerolidol	1558	45.47	±	28.54	d	138.63	±	13.02	c	130.49	±	35.21	c	222.80	±	75.63	ab	181.86	±	28.52	bc	232.61	±	37.66	ab	256.27	±	47.35	a
**46**	Bisabolol oxide II	1660	1.13	±	0.23	c	2.92	±	0.69	b	3.31	±	1.14	b	5.19	±	1.33	a	5.57	±	1.02	a	6.34	±	0.96	a	6.13	±	1.86	a
**47**	L-α-Bisabolol	1688	0.00	±	0.00	b	1.14	±	0.87	a	0.00	±	0.00	b	0.00	±	0.00	b	0.00	±	0.00	b	0.00	±	0.00	b	0.00	±	0.00	b
	***Sum of sesquiterpene alcohols***		46.60	±	28.71	e	142.70	±	13.46	cd	133.80	±	36.05	d	227.99	±	76.59	ab	187.44	±	29.34	bc	238.95	±	38.14	ab	262.39	±	47.83	a
	***Totals***		428.29	±	62.77	d	596.90	±	40.51	d	620.67	±	74.77	d	896.48	±	249.07	c	876.25	±	120.80	c	1218.06	±	185.96	b	1912.33	±	266.36	a

Values are mean ± standard deviation of three replicates. Within each column, different letters indicate significant differences among values (P< 0.05) using ANOVA followed by Tukey’s HSD test.

Prior to confirm the numerical results, the percentage change of each VOC group was investigated. Regardless of the treatments and storage periods, some groups including monoterpene, sesquiterpenes, acids, lactones, and ketones did not exceed 5% in percentage content (**Supplementary Figure 2**). Therefore, the total VOCs content was determined by aldehydes, esters, furans, monoterpene alcohols, and sesquiterpene alcohols. To visualize the variability of compound groups following the storage duration, regression model drawn.

**Figure 4:**
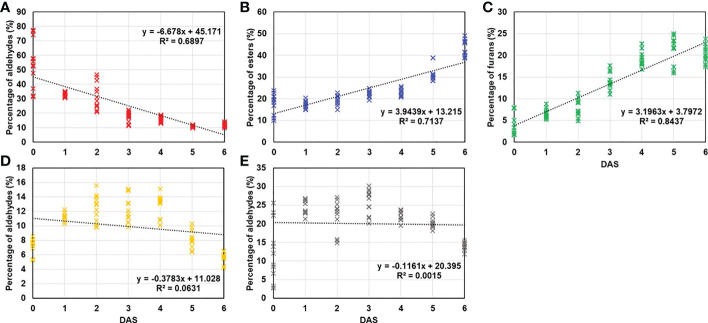
Regression analysis was conducted between DAS and percentage of main VOCs groups (over 5% of total VOCs) including **(A)** aldehydes, **(B)** esters, **(C)** furans, **(D)** monoterpene alcohols, and **(E)** sesquiterpene alcohols and regression analysis was conducted and represented by coefficient of determination (*R^2^*).

As shown in **Figure 4**, coefficient of determination (*R^2^*) in monoterpene alcohols (*R^2^* = 0.0631; **Figure 4D**) and sesquiterpene alcohols (*R^2^* = 0.0015; **Figure 4E**) explained the low variability across duration. Meanwhile, aldehydes (*R^2^* = 0.6897; **Figure 4A**), esters (*R^2^* = 0.7137; **Figure 4B**) and furans (*R^2^* = 0.8437; **Figure 4C**) exhibited strong correlation. Even in the compound groups that showed high correlation with storage period, the complex physiological processes result in the different tendency. It has been well documented that the aldehydes are produced from diverse precursors during storage and subsequent aldehydes decomposition contributes to esters and alcohols formation (Yan et al., 2018; Lu et al., 2020).

**Figure 5:**
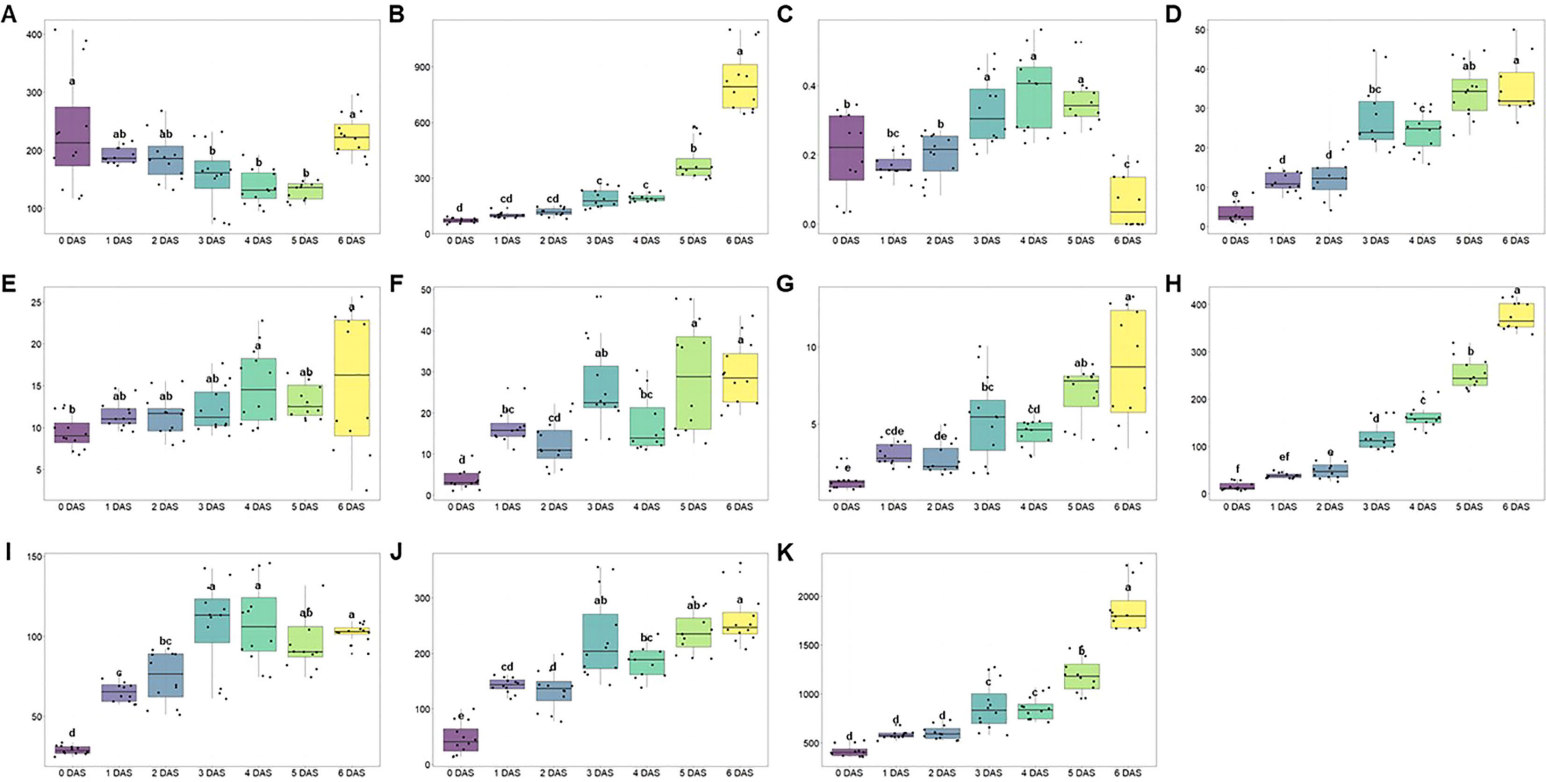
Box plots of **(A)** aldehydes, **(B)** esters, **(C)** monoterpene, **(D)** sesquiterpenes, **(E)** acids, **(F)** lactones, **(G)** ketones, **(H)** furans, **(I)** monoterpene alcohols, **(J)** sesquiterpene alcohols, and **(K)** total amounts following the days after storage (DAS) at room temperature (25°C). Different lowercase letters (a-e) indicate significant differences in the course of storage. DAS, days after storage.

**Figure 6:**
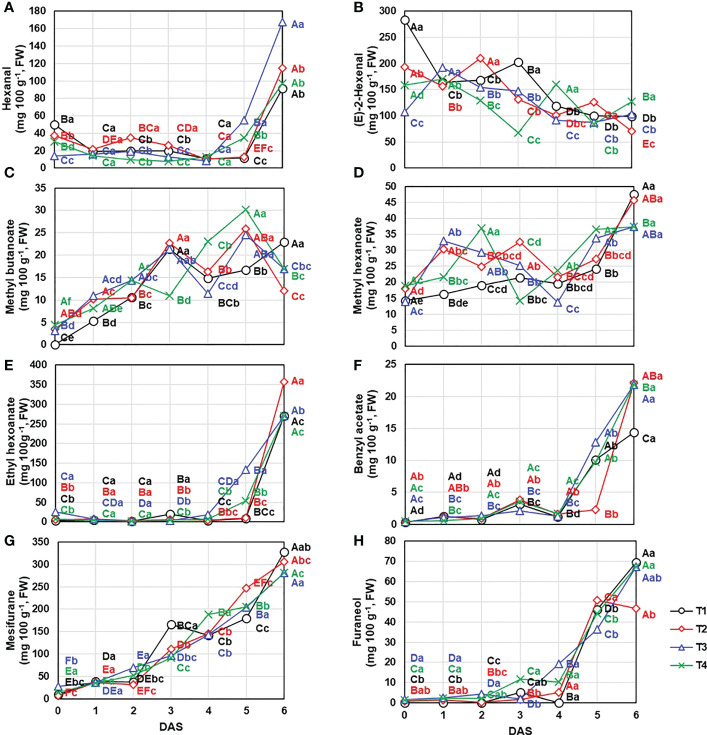
Content change of the important compounds contributing to main VOCs groups including **(A)** hexanal, **(B) (E)**-2-hexenal, **(C)** methyl butanoate, **(D)** methyl hexanoate, **(E)** ethyl hexanoate, **(F)** benzyl acetate, **(G)** mesifuran, and **(H)** furaneol under different drainage ratio treatments. Different capital letters **(A–C)** indicate significant differences under same drainage ratio treatments and lowercase letters (a, b) indicate significant differences among treatments in the course of storage. Drainage ratios represented as following: T1 (12.0%), T2 (25.3%), T3 (36.4%), and T4 (56.5%). DAS, days after storage.

These reported trends were agreement with our results. First, aldehydes were mainly composed of hexanal and (E)-2-hexenal, which accounted for 6.6 – 60.6% and 37.2 – 91.8% of total aldehydes content, the content of other compounds was low. The total aldehydes content showed decreased until 5 DAS (**Figure 5A**) following decline tendency of (E)-2-hexenal (0 DAS: 186.11 ± 67.49 mg 100 g^-1^; 1 DAS: 171.14 ± 15.39 mg 100 g^-1^; 2 DAS: 165.78 ± 31.62 mg 100 g^-1^; 3 DAS: 137.28 ± 50.95 mg 100 g^-1^; 4 DAS: 117.81 ± 28.38 mg 100 g^-1^; 5 DAS: 99.50 ± 18.51 mg 100 g^-1^; 6 DAS: 100.19 ± 21.59 mg 100 g^-1^, respectively) (**Figure 6B**). But total aldehydes content was increased at 6 DAS along with the hexanal, one of the main aldehydes of strawberry fruit, exhibited increase trend with the extension of the storage period (0 DAS: 32.86 ± 13.76 mg 100 g^-1^; 1 DAS: 17.74 ± 4.05 mg 100 g^-1^; 2 DAS: 20.62 ± 10.25 mg 100 g^-1^; 3 DAS: 16.69 ± 7.53 mg 100 g^-1^; 4 DAS: 10.67 ± 2.78 mg 100 g^-1^; 5 DAS: 28.48 ± 18.97 mg 100 g^-1^; 6 DAS: 117.82 ± 32.33 mg 100 g^-1^, respectively) (**Figure 6A**). In detail, there was no significant (*P*< 0.05) difference on hexanal content following the treatments until 4 DAS, but a significant difference appeared from the 5 DAS (*P*< 0.05) (T1: 11.58 ± 1.16 mg 100 g^-1^; T2: 12.58 ± 0.36 mg 100 g^-1^; T3: 55.06 ± 2.74 mg 100 g^-1^; T4: 34.69 ± 6.74 mg 100 g^-1^, respectively).

Meanwhile, esters and furans, which are the products of degradation of aldehydes, ascribed the positive change following the storage (**Figure 5B**). In case of esters, total 25 compounds were identified (**Table 3**) and the most important odorants of strawberry including methyl butanoate, methyl hexanoate, and ethyl hexanoate were detected (Yan et al., 2018). Consistent with previous reports (Zhang et al., 2023), the total content of esters showed a gradual increase (0 DAS: 72.50 ± 13.15 mg 100 g^-1^; 1 DAS: 101.27 ± 15.44 mg 100 g^-1^; 2 DAS: 117.62 ± 22.80 mg 100 g^-1^; 3 DAS: 190.12 ± 48.08 mg 100 g^-1^; 4 DAS: 195.35 ± 20.38 mg 100 g^-1^; 5 DAS: 388.16 ± 107.98 mg 100 g^-1^; 6 DAS: 827.50 ± 172.38 mg 100 g^-1^, respectively). Especially, total esters amount was almost doubled every day between 4 DAS and 6 DAS, so total amount of 6 DAS was about 4.30 times higher than that of 4 DAS. But following the compounds, their contribution to total contents change was different. Methyl butanoate and methyl hexanoate, which are known as the main components of esters, presented coefficient of determination (*R^2^*) of 0.1442 and 0.5753, respectively. So, these compounds content change was not consistent with total esters (**Figures 6C, D**). But, in this period from 4 DAS to 6 DAS, among main esters, ethyl hexanoate exhibited different change trend following the treatments (**Figure 6E**). Compared to 3 DAS, ethyl hexanoate content on 4 DAS decreased in T1 (0.16 times) and T2 (0.65 times) but increased in T3 (5.12 times) and T4 (1.48 times). These differences were more pronounced on 5 DAS through the increase ratio compared to 3 DAS. T3 and T4 rapidly increased by 38.12 and 11.99 times, but T1 and T2 exhibited low change ratios of 0.41 and 1.88 times, respectively. Addition to ethyl hexanoate, benzyl acetate (**Figure 6F**) also ascribed to have similar patterns. It also reported to increase during the post-harvest period and related to the anthocyanin biosynthesis pathway (Fu et al., 2017). T2 group, which presented the lowest anthocyanins content on 4 DAS as illustrated in **Figure 2**, showed the lowest benzyl acetate content on 5 DAS.

Not only esters but also furans showed a tendency to increase as the storage period is increased (**Figure 5H**). Two furan compounds (mesifuran and furaneol) were detected in ‘Seolhyang’ cultivar and in particular, regardless of storage period and treatment mesifuran was found to account for more than 77% of the total furans content so ascribed very strong correlation with 0.9923 of *R^2^*. While mesifuran (0 DAS: 14.65 ± 8.41 mg 100 g^-1^;1 DAS: 36.88 ± 5.78 mg 100 g^-1^; 2 DAS: 47.86 ± 16.01 mg 100 g^-1^; 3 DAS: 116.20 ± 31.44 mg 100 g^-1^; 4 DAS: 154.66 ± 22.82 mg 100 g^-1^; 5 DAS: 208.87 ± 27.76 mg 100 g^-1^; 6 DAS: 315.77 ± 28.24 mg 100 g^-1^, respectively) increased steadily during the storage period (**Figure 6G**), furaneol, the other minor compound of furans, was dramatically changed from 4 DAS (8.78 ± 7.68 mg 100 g^-1^) to 5 DAS (44.26 ± 7.79 mg 100 g^-1^) (**Figure 6H**).

## 4 Discussion

Strawberry is one of the highly perishable non-climacteric fruits, and the fruits must be harvested on maturity immediately (Cordenunsi et al., 2003; Hernandez-Munoz et al., 2008). Efforts have been made to overcome the low storability of strawberries (de Resende et al., 2008), and in this study, drain ratios influencing the water supply and fruit yield (Zahedi et al., 2020) were applied as the pretreatment.

Firmness, which can indicate a decrease in the degree of pectin esterification, has been used to evaluate effective methods of enhancing shelf life (Duvetter et al., 2005). As presented in **Figure 2B**, the firmness was consistently decreased and T2 and T3 exhibited significantly lower firmness than T1 and T4 at 6 DAS. Therefore, it was suggested that the drainage ratios could affect the storage capacity of strawberries, and metabolomic analyzes were conducted to support this.

At the harvest time (0 DAS), there was no significant differences in firmness, TSS (**Figure 2**), and functional components, anthocyanins (C3G and Pg3G) content (**Figures 3A, B**), but the antioxidant activity was different (**Figure 3C**). As the drainage ratio increased, the antioxidant activity decreased and it affected many mechanisms afterward. The low antioxidant activity could stimulate the decomposition of cellular structure and leads to senescence (Jiang and Joyce, 2003; Wu et al., 2018). And induced senescence increases the levels of anthocyanin until the mature stage then, declines during over-ripening (da Silva et al., 2007; Karaaslan and Yaman, 2017). The T1 and T2, which showed significantly higher antioxidant activities than T3 and T4, appeared a peak of anthocyanin content on 5 DAS (fully-mature stage) and declined at the 6 DAS (over-mature stage showing decay) with relatively delayed over-ripening rates compared to T3 and T4 (**Figure 2**), which was consistent with the previous studies (Ayala-Zavala et al., 2004; Woods et al., 2005). Addition to the anthocyanins accumulation, the degradation of cell wall induced by oxygen stress results in the emitting of VOCs (Woods et al., 2005).

Therefore, volatilomics analysis of pretreated strawberry under the different drainage ratios was conducted during storage for evaluating the VOCs profiling and their distribution (**Figures 5A–K**). A total of 47 compounds were detected by HS-SPME analysis (**Table 3**), and their patterns were different under various drainage treatments.

Among these compound groups, the aldehydes, esters and furans, which accounted for more than 5% in percentage content, were expected as the prominent compounds influencing on the strawberry flavor (Larsen and Poll, 1992; Forney et al., 2000; Rey-Serra et al., 2022). While the fruit senescence, VOCs are responsible for esterification of alcohols, and degradation of amino acids and fatty acids (Severo et al., 2011). Especially, the accumulation of lipid peroxide during storage derives aldehydes production (Lu et al., 2020) through oxidative degradation of lipoxygenase (LOX) or hydroperoxide lyase (HPL) (Yan et al., 2018). And alcohol dehydrogenase gene (ADH) contributing in converting of aldehydes to alcohols and alcohol acyltransferase gene (AAT) involving in the esterification process (Lu et al., 2020; Zhang et al., 2023) encode gene in the biosynthetic pathway of esters.

Aldehydes, which are high concentrations in immature fruits presenting green notes in the early stages of ripening, decreased during the storage (Ozcan and Barringer, 2011). And this decreasing tendency of aldehyde (**Figure 5A**) was determined by (E)-2-hexenal (**Figure 6B**) due to a high occupancy of it making up a proportion of 79.0% of total aldehydes content (**Table 3**) but the trend of some aldehydes such as hexanal (decay indicator) was not consistent with this (**Figure 6A**). Meanwhile, esters showed opposite trend with it. The ester-based components (butyl acetate, hexyl acetate, benzyl acetate, methyl butanoate, ethyl butanoate, butyl butanoate, methyl hexanoate, and ethyl hexanoate) offering the fruity and sweet notes of strawberries are commonly identified (Forney et al., 2000; Rey-Serra et al., 2022). In case of “Seolhyang” variety, mainly dominated by the acetic-, butanoic-, hexanoic-, and octanoic-acid derived esters, and well reported esters, methyl hexanoate and ethyl hexanoate, were observed. As described in **Figure 5B**, total esters content was consistently increased during the storage, and synthesis rate of ethyl hexanoate, one of the main esters, was delayed at T1 and T2 (**Figure 6E**). Interestingly, the delayed trend of a peak of anthocyanin accumulation and the emission of the over-ripening indicators, hexanal and ethyl hexanoate, in T1 and T2, indicated somewhat better storability. According to the study of Severo et al. (2011), the expression level of phenylamine ammonia lyase (PAL) and anthocyanidin synthase (ANS), which are involving in anthocyanins biosynthesis pathway, drastically increased at specific developmental stage, which coincided with the point of increase in AAT expression. Even the up-regulated PAL leads the increase of benzyl acetate. It has been reported to be synthesized through shikimate pathway as using L-phenylalanine as the precursor (Kojima et al., 2015). As the PAL is involved in upper biosynthesis pathway of benzyl acetate, the anthocyanins accumulation trend (T3 exhibited the highest content but the lowest content was quantified in T2) was agreement with benzyl acetate content. When data (content of aldehydes and esters) are collected proportionally, regardless of the treatments, the ratio was decreased consistently as the storage period, and it can be used as the evaluation of strawberry quality during the storage.

Furanones are the most well-known furan compounds relevant in strawberry flavor by imparting caramel and sweet notes (Larsen and Poll, 1992). Furaneol (DHF, 4-hydroxy-2,5-dimethylfuran-3-one) and mesifuran (DMF, 4-methoxy-2,5-dimethylfuran-3-one) are the furanones that are most generally reported. These are generally found in all strawberry varieties and are considered as the typical impact aroma of strawberries (Mënager et al., 2004). These are key aroma-active VOCs in strawberries, are responsible for ripening development, so they reach the peak at the overripe stage (Përez et al., 1996).

This present study was aimed at pointing toward VOCs as the shelf-life indicators of strawberry and determining proper drainage ratio based on these as well as firmness, TSS, anthocyanins, and antioxidant activity. Among identified VOCs, accumulation of stress- and over-ripening-related VOCs (hexanal and ethyl hexanoate) presented the possibility to be used as a reference in the practical fields. And based on these, T2 was the most efficient drainage condition with the positive effects on saving water along with securing yield during preharvest and showing lower levels of senescence-related metabolites. Thus, drainage ratio in the preharvest step might be a promising approach for postharvest of horticultural product.

## Data availability statement

The original contributions presented in the study are included in the article/**Supplementary Material**. Further inquiries can be directed to the corresponding author.

## Author contributions

JC: Conceptualization, Methodology, and Writing-original draft. DR: Conceptualization and Methodology. MH: Writing-review and editing. H-YK: Conceptualization, Data curation, Writing-review and editing, Supervision. All authors contributed to the article and approved the submitted version.
