# Supplementary material for: Effect of drainage ratio during strawberry cultivation:The volatilomics-based shelf-life indicators for strawberry fruit
Source: Front Plant Sci. 2023 Mar 21;14:1124827. doi: 10.3389/fpls.2023.1124827 (PMC10070737; doi:10.3389/fpls.2023.1124827)
Supplement: Supplementary file 1 [file DataSheet_1.docx]

**Supplementary data**

**Supplementary Table 1.** Profiling of volatile organic compounds (VOCs) in strawberry fruits during the storage (mg 100g^-1^) (continues…)

| **Compound name** | | **RI** | **Storage time (days)** | | | | | | | | | | | | | | | | | | | | | | | | |
| --- | --- | --- | --- | --- | --- | --- | --- | --- | --- | --- | --- | --- | --- | --- | --- | --- | --- | --- | --- | --- | --- | --- | --- | --- | --- | --- | --- |
|  |  |  | **0 d** | | | | | | | | | | | |  | **1 d** | | | | | | | | | | | |
|  |  |  | **10.6%** | | | **21.2%** | | | **35.0%** | | | **54.4%** | | |  | **10.6%** | | | **21.2%** | | | **35.0%** | | | **54.4%** | | |
| *Aldehydes* | |  |  |  |  |  |  |  |  |  |  |  |  |  |  |  |  |  |  |  |  |  |  |  |  |  |  |
| 1 | Hexanal | 804 | 50.08 | ± | 1.83 | 37.15 | ± | 1.71 | 13.97 | ± | 2.64 | 30.24 | ± | 3.16 |  | 19.38 | ± | 1.83 | 21.60 | ± | 3.23 | 16.01 | ± | 2.13 | 13.99 | ± | 4.45 |
| 2 | (E)-2-Hexenal | 854 | 284.16 | ± | 12.26 | 193.55 | ± | 8.41 | 107.9 | ± | 5.58 | 158.83 | ± | 1.82 |  | 164.57 | ± | 8.49 | 156.68 | ± | 4.04 | 192.45 | ± | 7.45 | 170.88 | ± | 9.99 |
| 3 | Heptanal | 901 | 52.6 | ± | 3.70 | 0.44 | ± | 0.03 | 0.29 | ± | 0.01 | 0.30 | ± | 0.04 |  | 0.39 | ± | 0.06 | 0.60 | ± | 0.02 | 0.32 | ± | 0.07 | 0.39 | ± | 0.04 |
| 4 | (E,E)-2,4-Hexadienal | 908 | 1.48 | ± | 0.12 | 1.18 | ± | 0.14 | 0.47 | ± | 0.26 | 0.59 | ± | 0.25 |  | 0.31 | ± | 0.03 | 0.64 | ± | 0.14 | 0.44 | ± | 0.02 | 0.48 | ± | 0.03 |
| 5 | Benzaldehyde | 963 | 0.15 | ± | 0.21 | 0.06 | ± | 0.02 | 0.09 | ± | 0.05 | 0.28 | ± | 0.06 |  | 0.30 | ± | 0.04 | 0.29 | ± | 0.22 | 0.20 | ± | 0.06 | 0.27 | ± | 0.09 |
| 6 | Octanal | 1005 | 0.75 | ± | 0.14 | 0.64 | ± | 0.19 | 0.59 | ± | 0.06 | 0.49 | ± | 0.08 |  | 0.42 | ± | 0.05 | 0.77 | ± | 0.07 | 0.47 | ± | 0.12 | 0.57 | ± | 0.16 |
| 7 | Decanal | 1205 | 0.76 | ± | 0.21 | 0.79 | ± | 0.07 | 0.44 | ± | 0.07 | 0.75 | ± | 0.07 |  | 1.10 | ± | 0.18 | 1.11 | ± | 0.08 | 0.50 | ± | 0.09 | 0.64 | ± | 0.11 |
| ***Sum of aldehydes*** | | | 389.98 | ± | 16.87 | 233.83 | ± | 6.84 | 123.76 | ± | 7.92 | 191.47 | ± | 4.83 |  | 186.48 | ± | 6.96 | 181.68 | ± | 7.21 | 210.38 | ± | 6.26 | 187.22 | ± | 14.67 |
| *Esters* | |  |  |  |  |  |  |  |  |  |  |  |  |  |  |  |  |  |  |  |  |  |  |  |  |  |  |
| 8 | Methyl butanoate | 723 | 0.00 | ± | 0.00 | 3.44 | ± | 0.57 | 3.16 | ± | 0.58 | 4.42 | ± | 0.50 |  | 5.38 | ± | 1.44 | 10.21 | ± | 1.71 | 10.99 | ± | 1.43 | 8.17 | ± | 0.78 |
| 9 | Methyl 2-methylbutyrate | 785 | 0.74 | ± | 0.43 | 0.33 | ± | 0.02 | 0.16 | ± | 0.11 | 0.30 | ± | 0.12 |  | 0.15 | ± | 0.04 | 0.39 | ± | 0.18 | 0.57 | ± | 0.06 | 0.44 | ± | 0.07 |
| 10 | Isopropyl butyrate | 846 | 0.03 | ± | 0.02 | 0.03 | ± | 0.01 | 0.02 | ± | 0.01 | 0.05 | ± | 0.02 |  | 0.23 | ± | 0.09 | 0.39 | ± | 0.01 | 0.33 | ± | 0.05 | 0.29 | ± | 0.13 |
| 11 | Methyl hexanoate | 926 | 14.28 | ± | 1.37 | 18.07 | ± | 3.42 | 13.86 | ± | 3.3 | 18.96 | ± | 0.40 |  | 16.23 | ± | 0.99 | 30.46 | ± | 2.31 | 32.87 | ± | 3.80 | 21.71 | ± | 3.71 |
| 12 | Ethyl hexanoate | 998 | 2.63 | ± | 1.00 | 6.68 | ± | 3.36 | 25.65 | ± | 3.83 | 7.89 | ± | 0.30 |  | 4.13 | ± | 2.78 | 7.80 | ± | 1.30 | 7.68 | ± | 8.75 | 4.35 | ± | 2.56 |
| 13 | Hexyl acetate | 1011 | 5.86 | ± | 0.71 | 9.88 | ± | 1.63 | 3.98 | ± | 0.17 | 3.22 | ± | 0.89 |  | 4.97 | ± | 3.50 | 2.80 | ± | 0.76 | 6.76 | ± | 0.18 | 5.52 | ± | 0.70 |
| 14 | 2-Hexen-1-yl acetate | 1019 | 12.76 | ± | 4.99 | 24.68 | ± | 3.25 | 11.11 | ± | 0.86 | 8.43 | ± | 1.62 |  | 15.04 | ± | 3.73 | 5.56 | ± | 0.73 | 20.69 | ± | 1.97 | 19.53 | ± | 6.21 |
| 15 | Methyl octanoate | 1026 | 0.30 | ± | 0.06 | 0.61 | ± | 0.18 | 0.75 | ± | 0.25 | 0.72 | ± | 0.21 |  | 0.87 | ± | 0.07 | 1.20 | ± | 0.22 | 1.29 | ± | 0.30 | 0.93 | ± | 0.26 |
| 16 | Benzyl acetate | 1164 | 1.71 | ± | 0.43 | 2.12 | ± | 0.54 | 1.55 | ± | 0.4 | 3.78 | ± | 0.78 |  | 9.87 | ± | 3.16 | 6.55 | ± | 1.24 | 12.14 | ± | 1.78 | 12.8 | ± | 2.60 |
| 17 | Ethyl benzoate | 1172 | 0.32 | ± | 0.03 | 0.31 | ± | 0.03 | 0.37 | ± | 0.02 | 0.48 | ± | 0.21 |  | 1.27 | ± | 0.48 | 1.16 | ± | 0.12 | 1.11 | ± | 0.12 | 0.54 | ± | 0.02 |
| 18 | 2-(2-Butoxyethoxy)ethanol | 1192 | 5.49 | ± | 0.94 | 7.28 | ± | 1.18 | 4.17 | ± | 0.04 | 4.98 | ± | 0.75 |  | 6.19 | ± | 0.36 | 8.38 | ± | 0.40 | 6.40 | ± | 0.87 | 6.64 | ± | 0.93 |
| 19 | Ethyl octanoate | 1194 | 0.12 | ± | 0.02 | 0.25 | ± | 0.11 | 1.83 | ± | 0.41 | 0.50 | ± | 0.13 |  | 0.17 | ± | 0.09 | 0.18 | ± | 0.09 | 0.21 | ± | 0.09 | 0.44 | ± | 0.51 |
| 20 | Methyl salicylate | 1195 | 4.20 | ± | 1.06 | 4.56 | ± | 0.42 | 3.18 | ± | 0.40 | 5.57 | ± | 0.36 |  | 7.83 | ± | 0.76 | 3.56 | ± | 1.51 | 2.20 | ± | 0.39 | 2.70 | ± | 0.21 |
| 21 | Octyl acetate | 1211 | 0.40 | ± | 0.32 | 0.42 | ± | 0.07 | 0.98 | ± | 0.14 | 0.61 | ± | 0.12 |  | 1.82 | ± | 0.63 | 1.59 | ± | 0.65 | 1.29 | ± | 0.37 | 1.04 | ± | 0.22 |
| 22 | 2-Nonanol, acetate | 1236 | 0.15 | ± | 0.06 | 0.10 | ± | 0.04 | 0.37 | ± | 0.09 | 0.19 | ± | 0.13 |  | 0.67 | ± | 0.14 | 0.69 | ± | 0.09 | 1.13 | ± | 0.56 | 0.81 | ± | 0.08 |
| 23 | Isopentyl hexanoate | 1245 | 0.16 | ± | 0.07 | 0.10 | ± | 0.06 | 0.15 | ± | 0.01 | 0.53 | ± | 0.66 |  | 0.34 | ± | 0.06 | 0.18 | ± | 0.05 | 0.19 | ± | 0.05 | 0.10 | ± | 0.03 |
| 24 | Methyl 3-hydroxyoctanoate | 1253 | 0.49 | ± | 0.26 | 0.20 | ± | 0.02 | 2.27 | ± | 0.35 | 1.54 | ± | 1.99 |  | 2.24 | ± | 0.84 | 2.27 | ± | 0.43 | 1.04 | ± | 0.39 | 2.74 | ± | 1.43 |
| 25 | Triacetin | 1346 | 0.42 | ± | 0.05 | 0.41 | ± | 0.05 | 0.28 | ± | 0.04 | 0.33 | ± | 0.04 |  | 0.47 | ± | 0.01 | 0.58 | ± | 0.10 | 0.48 | ± | 0.06 | 0.55 | ± | 0.10 |
| 26 | 2-(2-Butoxyethoxy)ethyl acetate | 1366 | 6.12 | ± | 0.93 | 6.28 | ± | 0.25 | 4.11 | ± | 0.36 | 4.54 | ± | 0.17 |  | 6.16 | ± | 0.36 | 6.80 | ± | 0.35 | 7.56 | ± | 0.83 | 8.31 | ± | 0.16 |
| 27 | Octyl 2-methylbutyrate | 1442 | 0.19 | ± | 0.10 | 0.12 | ± | 0.02 | 0.49 | ± | 0.07 | 0.21 | ± | 0.07 |  | 1.02 | ± | 0.34 | 0.77 | ± | 0.07 | 0.73 | ± | 0.14 | 0.63 | ± | 0.12 |
| 28 | Acetic acid, cinnamyl ester | 1449 | 0.03 | ± | 0.01 | 0.06 | ± | 0.03 | 0.13 | ± | 0.05 | 0.16 | ± | 0.08 |  | 3.19 | ± | 1.32 | 2.50 | ± | 0.63 | 2.68 | ± | 0.67 | 1.90 | ± | 0.47 |
| 29 | Octyl hexanoate | 1575 | 0.17 | ± | 0.21 | 0.04 | ± | 0.02 | 1.24 | ± | 0.73 | 0.20 | ± | 0.11 |  | 0.86 | ± | 0.24 | 0.72 | ± | 0.12 | 1.74 | ± | 2.42 | 1.00 | ± | 0.26 |
|  | ***Sum of esters*** | | 56.57 | ± | 4.95 | 85.99 | ± | 6.35 | 79.81 | ± | 8.23 | 67.63 | ± | 7.06 |  | 89.10 | ± | 3.14 | 94.75 | ± | 7.50 | 120.09 | ± | 17.10 | 101.13 | ± | 11.63 |
| *Monoterpene* | |  |  |  |  |  |  |  |  |  |  |  |  |  |  |  |  |  |  |  |  |  |  |  |  |  |  |
| 30 | β-Ocimene | 1049 | 0.31 | ± | 0.04 | 0.30 | ± | 0.03 | 0.16 | ± | 0.02 | 0.04 | ± | 0.01 |  | 0.19 | ± | 0.03 | 0.16 | ± | 0.05 | 0.15 | ± | 0.02 | 0.17 | ± | 0.03 |
|  | ***Sum of monoterpenes*** | | 0.31 | ± | 0.04 | 0.30 | ± | 0.03 | 0.16 | ± | 0.02 | 0.04 | ± | 0.01 |  | 0.19 | ± | 0.03 | 0.16 | ± | 0.05 | 0.15 | ± | 0.02 | 0.17 | ± | 0.03 |
| Sesquiterpenes | |  |  |  |  |  |  |  |  |  |  |  |  |  |  |  |  |  |  |  |  |  |  |  |  |  |  |
| 31 | (Z)-β-Famesene | 1442 | 0.62 | ± | 0.31 | 1.31 | ± | 0.20 | 4.33 | ± | 0.74 | 1.85 | ± | 0.30 |  | 7.74 | ± | 1.24 | 6.40 | ± | 2.41 | 6.31 | ± | 1.37 | 6.57 | ± | 0.76 |
| 32 | (E)-β-Farnesene | 1459 | 0.19 | ± | 0.05 | 0.33 | ± | 0.06 | 1.03 | ± | 0.23 | 0.47 | ± | 0.11 |  | 1.93 | ± | 0.3 | 1.54 | ± | 0.18 | 1.85 | ± | 0.65 | 1.84 | ± | 0.51 |
| 33 | (Z,E)-α-Farnesene | 1479 | 0.25 | ± | 0.11 | 0.44 | ± | 0.07 | 1.72 | ± | 0.34 | 1.05 | ± | 0.78 |  | 3.16 | ± | 0.54 | 2.40 | ± | 1.53 | 3.01 | ± | 0.53 | 2.65 | ± | 0.41 |
|  | ***Sum of sesquiterpenes*** | | 1.06 | ± | 0.45 | 2.08 | ± | 0.32 | 7.07 | ± | 1.30 | 3.36 | ± | 1.18 |  | 12.83 | ± | 2.08 | 10.34 | ± | 4.02 | 11.17 | ± | 2.45 | 11.05 | ± | 1.68 |
| *Acids* | |  |  |  |  |  |  |  |  |  |  |  |  |  |  |  |  |  |  |  |  |  |  |  |  |  |  |
| 34 | 2-Ethylhexanoic acid | 1125 | 0.98 | ± | 0.29 | 0.92 | ± | 0.1 | 0.59 | ± | 0.09 | 0.59 | ± | 0.02 |  | 0.67 | ± | 0.08 | 0.70 | ± | 0.12 | 0.73 | ± | 0.05 | 0.75 | ± | 0.05 |
| 35 | Nonanoic acid | 1276 | 8.55 | ± | 2.57 | 9.60 | ± | 1.53 | 7.43 | ± | 1.11 | 9.30 | ± | 2.44 |  | 10.74 | ± | 0.94 | 11.08 | ± | 2.58 | 9.98 | ± | 0.34 | 11.5 | ± | 2.38 |
| 36 | Hydrocinnamic acid | 1350 | 0.00 | ± | 0.00 | 0.00 | ± | 0.00 | 0.00 | ± | 0.00 | 0.00 | ± | 0.00 |  | 0.00 | ± | 0.00 | 0.00 | ± | 0.00 | 0.00 | ± | 0.00 | 0.00 | ± | 0.00 |
|  | ***Sum of acids*** | | 9.52 | ± | 2.84 | 10.53 | ± | 1.59 | 8.02 | ± | 1.10 | 9.90 | ± | 2.45 |  | 11.40 | ± | 1.02 | 11.77 | ± | 2.64 | 10.71 | ± | 0.30 | 12.25 | ± | 2.41 |
| *Lactone* | |  |  |  |  |  |  |  |  |  |  |  |  |  |  |  |  |  |  |  |  |  |  |  |  |  |  |
| 37 | γ-Dodecalactone | 1472 | 1.96 | ± | 1.34 | 2.66 | ± | 0.49 | 4.56 | ± | 1.62 | 6.12 | ± | 3.28 |  | 18.55 | ± | 2.53 | 19.46 | ± | 5.66 | 15.17 | ± | 1.53 | 13.12 | ± | 1.78 |
|  | ***Sum of lactones*** | | 1.96 | ± | 1.34 | 2.66 | ± | 0.49 | 4.56 | ± | 1.62 | 6.12 | ± | 3.28 |  | 18.55 | ± | 2.53 | 19.46 | ± | 5.66 | 15.17 | ± | 1.53 | 13.12 | ± | 1.78 |
| *Ketone* | |  |  |  |  |  |  |  |  |  |  |  |  |  |  |  |  |  |  |  |  |  |  |  |  |  |  |
| 38 | 2-Heptanone | 891 | 0.78 | ± | 0.10 | 1.21 | ± | 0.23 | 2.11 | ± | 0.71 | 1.13 | ± | 0.22 |  | 2.31 | ± | 0.26 | 3.97 | ± | 0.17 | 2.89 | ± | 0.4 | 2.95 | ± | 0.60 |
|  | ***Sum of ketones*** | | 0.78 | ± | 0.10 | 1.21 | ± | 0.23 | 2.11 | ± | 0.71 | 1.13 | ± | 0.22 |  | 2.31 | ± | 0.26 | 3.97 | ± | 0.17 | 2.89 | ± | 0.4 | 2.95 | ± | 0.60 |
| *Furans* | |  |  |  |  |  |  |  |  |  |  |  |  |  |  |  |  |  |  |  |  |  |  |  |  |  |  |
| 39 | Mesifuran | 1067 | 9.50 | ± | 1.17 | 7.35 | ± | 2.24 | 27.49 | ± | 1.16 | 14.27 | ± | 3.82 |  | 38.76 | ± | 5.72 | 36.96 | ± | 9.9 | 36.58 | ± | 5.18 | 35.21 | ± | 4.04 |
| 40 | Furaneol | 1081 | 0.15 | ± | 0.01 | 1.01 | ± | 0.25 | 1.80 | ± | 0.14 | 1.52 | ± | 0.65 |  | 0.22 | ± | 0.10 | 1.39 | ± | 0.59 | 2.61 | ± | 0.94 | 2.25 | ± | 0.39 |
|  | ***Sum of furans*** | | 9.65 | ± | 1.17 | 8.36 | ± | 2.03 | 29.29 | ± | 1.04 | 15.79 | ± | 4.06 |  | 38.98 | ± | 5.72 | 38.35 | ± | 9.80 | 39.19 | ± | 5.81 | 37.46 | ± | 4.42 |
| *Monoterpene alcohols* | |  |  |  |  |  |  |  |  |  |  |  |  |  |  |  |  |  |  |  |  |  |  |  |  |  |  |
| 41 | (E)-Linalool oxide | 1086 | 2.83 | ± | 1.22 | 2.85 | ± | 0.17 | 2.15 | ± | 0.32 | 3.49 | ± | 0.35 |  | 3.18 | ± | 1.57 | 4.16 | ± | 0.65 | 4.58 | ± | 0.80 | 4.61 | ± | 0.64 |
| 42 | Linalool | 1099 | 22.33 | ± | 1.19 | 26.65 | ± | 2.19 | 24.43 | ± | 2.94 | 24.26 | ± | 1.87 |  | 57.08 | ± | 7.56 | 53.66 | ± | 5.16 | 63.8 | ± | 3.30 | 54.59 | ± | 6.00 |
| 43 | L-α-Terpineol | 1198 | 1.51 | ± | 0.14 | 1.69 | ± | 0.17 | 1.38 | ± | 0.55 | 1.53 | ± | 0.48 |  | 3.36 | ± | 0.30 | 4.7 | ± | 1.47 | 3.23 | ± | 0.28 | 3.22 | ± | 0.60 |
| 44 | Nerol | 1248 | 0.39 | ± | 0.11 | 0.21 | ± | 0.03 | 0.18 | ± | 0.01 | 0.24 | ± | 0.06 |  | 0.47 | ± | 0.09 | 0.64 | ± | 0.22 | 0.47 | ± | 0.10 | 0.53 | ± | 0.08 |
|  | ***Sum of monoterpene alcohols*** | | 27.06 | ± | 0.59 | 31.40 | ± | 2.01 | 28.14 | ± | 3.55 | 29.52 | ± | 2.43 |  | 64.09 | ± | 8.30 | 63.16 | ± | 5.88 | 72.08 | ± | 4.20 | 62.95 | ± | 5.90 |
| Sesquiterpene alcohols | |  |  |  |  |  |  |  |  |  |  |  |  |  |  |  |  |  |  |  |  |  |  |  |  |  |  |
| 45 | Nerolidol | 1558 | 13.96 | ± | 1.36 | 31.79 | ± | 5.57 | 86.13 | ± | 10.54 | 49.98 | ± | 7.40 |  | 145.83 | ± | 8.19 | 147.2 | ± | 7.88 | 140.15 | ± | 10.84 | 121.36 | ± | 6.86 |
| 46 | Bisabolol oxide II | 1660 | 0.99 | ± | 0.11 | 1.09 | ± | 0.15 | 1.43 | ± | 0.22 | 1.00 | ± | 0.15 |  | 2.22 | ± | 0.10 | 3.54 | ± | 0.88 | 2.94 | ± | 0.30 | 3.00 | ± | 0.66 |
| 47 | L-α-Bisabolol | 1688 | 0.00 | ± | 0.00 | 0.00 | ± | 0.00 | 0.00 | ± | 0.00 | 0.00 | ± | 0.00 |  | 0.95 | ± | 0.58 | 2.13 | ± | 0.38 | 1.49 | ± | 0.04 | 0.00 | ± | 0.00 |
|  | ***Sum of sesquiterpene alcohols*** | | 14.96 | ± | 1.46 | 32.88 | ± | 5.41 | 87.56 | ± | 10.75 | 50.98 | ± | 7.52 |  | 148.99 | ± | 7.83 | 152.86 | ± | 7.45 | 144.58 | ± | 10.71 | 124.36 | ± | 6.44 |
|  | ***Totals*** | | 525.64 | ± | 12.75 | 422.50 | ± | 7.94 | 379.08 | ± | 17.82 | 385.96 | ± | 23.26 |  | 585.15 | ± | 25.42 | 591.32 | ± | 25.11 | 641.68 | ± | 46.72 | 569.43 | ± | 34.25 |

(Continues…)

| **Compound name** | | **RT** | **Storage time (days)** | | | | | | | | | | | | | | | | | | | | | | | | | |  |
| --- | --- | --- | --- | --- | --- | --- | --- | --- | --- | --- | --- | --- | --- | --- | --- | --- | --- | --- | --- | --- | --- | --- | --- | --- | --- | --- | --- | --- | --- |
|  |  |  | **2 d** | | | | | | | | | | | |  | **3 d** | | | | | | | | | | | | |  |
|  |  |  | **10.6%** | | | **21.2%** | | | **35.0%** | | | **54.4%** | | |  | **10.6%** | | | **21.2%** | | | **35.0%** | | | **54.4%** | | |  |  |
| *Aldehydes* | |  |  |  |  |  |  |  |  |  |  |  |  |  |  |  |  |  |  |  |  |  |  |  |  |  |  |  |  |
| 1 | Hexanal | 804 | 19.44 | ± | 3.59 | 34.82 | ± | 4.35 | 18.87 | ± | 6.69 | 9.36 | ± | 1.38 |  | 19.91 | ± | 0.93 | 26.18 | ± | 4.17 | 12.87 | ± | 0.89 | 7.79 | ± | 1.06 |  |  |
| 2 | (E)-2-Hexenal | 854 | 168.53 | ± | 4.43 | 210.44 | ± | 12.74 | 154.49 | ± | 9.64 | 129.65 | ± | 8.42 |  | 203.32 | ± | 4.52 | 131.88 | ± | 8.36 | 146.76 | ± | 8.30 | 67.16 | ± | 4.92 |  |  |
| 3 | Heptanal | 901 | 0.39 | ± | 0.10 | 0.46 | ± | 0.12 | 0.47 | ± | 0.07 | 0.40 | ± | 0.05 |  | 0.52 | ± | 0.10 | 0.43 | ± | 0.01 | 0.3 | ± | 0.02 | 0.23 | ± | 0.03 |  |  |
| 4 | (E,E)-2,4-Hexadienal | 908 | 0.30 | ± | 0.07 | 0.83 | ± | 0.49 | 0.50 | ± | 0.13 | 0.22 | ± | 0.06 |  | 0.19 | ± | 0.03 | 0.38 | ± | 0.06 | 0.07 | ± | 0.01 | 0.11 | ± | 0.02 |  |  |
| 5 | Benzaldehyde | 963 | 0.09 | ± | 0.02 | 0.13 | ± | 0.04 | 0.19 | ± | 0.04 | 0.14 | ± | 0.07 |  | 0.85 | ± | 0.06 | 0.15 | ± | 0.03 | 0.32 | ± | 0.05 | 0.19 | ± | 0.15 |  |  |
| 6 | Octanal | 1005 | 0.32 | ± | 0.03 | 0.48 | ± | 0.10 | 0.68 | ± | 0.08 | 0.55 | ± | 0.10 |  | 0.38 | ± | 0.14 | 0.6 | ± | 0.21 | 0.38 | ± | 0.06 | 0.33 | ± | 0.01 |  |  |
| 7 | Decanal | 1205 | 0.99 | ± | 0.12 | 0.92 | ± | 0.35 | 1.47 | ± | 0.19 | 1.01 | ± | 0.12 |  | 1.66 | ± | 0.23 | 1.05 | ± | 0.10 | 0.76 | ± | 0.13 | 0.82 | ± | 0.04 |  |  |
|  | ***Sum of aldehydes*** | | 190.04 | ± | 7.41 | 248.08 | ± | 17.78 | 176.68 | ± | 15.77 | 141.33 | ± | 9.55 |  | 226.82 | ± | 4.74 | 160.66 | ± | 5.65 | 161.47 | ± | 8.88 | 76.64 | ± | 4.98 |  |  |
| *Esters* | |  |  |  |  |  |  |  |  |  |  |  |  |  |  |  |  |  |  |  |  |  |  |  |  |  |  |  | |
| 8 | Methyl butanoate | 723 | 10.56 | ± | 1.33 | 10.46 | ± | 0.41 | 14.34 | ± | 1.69 | 14.44 | ± | 1.34 |  | 21.38 | ± | 2.43 | 22.69 | ± | 0.85 | 21.39 | ± | 3.09 | 11.01 | ± | 1.12 |  |  |
| 9 | Methyl 2-methylbutyrate | 785 | 0.48 | ± | 0.04 | 0.56 | ± | 0.14 | 0.75 | ± | 0.24 | 0.61 | ± | 0.21 |  | 0.71 | ± | 0.14 | 0.89 | ± | 0.20 | 0.78 | ± | 0.08 | 1.55 | ± | 0.79 |  |  |
| 10 | Isopropyl butyrate | 846 | 0.39 | ± | 0.09 | 0.32 | ± | 0.01 | 0.50 | ± | 0.20 | 0.48 | ± | 0.11 |  | 0.45 | ± | 0.10 | 0.57 | ± | 0.08 | 0.56 | ± | 0.10 | 0.18 | ± | 0.01 |  |  |
| 11 | Methyl hexanoate | 926 | 19.02 | ± | 2.00 | 24.83 | ± | 5.68 | 29.4 | ± | 3.10 | 36.9 | ± | 0.77 |  | 21.52 | ± | 2.93 | 32.58 | ± | 2.55 | 25.18 | ± | 2.58 | 14.17 | ± | 1.48 |  |  |
| 12 | Ethyl hexanoate | 998 | 1.61 | ± | 0.70 | 3.51 | ± | 1.22 | 1.56 | ± | 0.26 | 1.89 | ± | 1.17 |  | 20.62 | ± | 3.07 | 6.21 | ± | 1.79 | 3.56 | ± | 0.63 | 4.64 | ± | 0.91 |  |  |
| 13 | Hexyl acetate | 1011 | 2.00 | ± | 0.70 | 4.14 | ± | 0.70 | 4.15 | ± | 0.77 | 3.29 | ± | 1.02 |  | 3.94 | ± | 0.71 | 5.52 | ± | 1.25 | 3.46 | ± | 0.35 | 4.98 | ± | 0.91 |  |  |
| 14 | 2-Hexen-1-yl acetate | 1019 | 6.04 | ± | 3.16 | 13.85 | ± | 1.07 | 14.59 | ± | 3.61 | 10.31 | ± | 4.59 |  | 4.65 | ± | 0.74 | 23.31 | ± | 6.11 | 9.36 | ± | 1.20 | 16.27 | ± | 1.60 |  |  |
| 15 | Methyl octanoate | 1026 | 0.91 | ± | 0.25 | 0.71 | ± | 0.12 | 0.89 | ± | 0.16 | 1.66 | ± | 0.09 |  | 1.44 | ± | 0.31 | 1.79 | ± | 0.26 | 1.04 | ± | 0.24 | 0.84 | ± | 0.17 |  |  |
| 16 | Benzyl acetate | 1164 | 16.01 | ± | 2.63 | 18.18 | ± | 3.79 | 32.2 | ± | 3.36 | 26.73 | ± | 3.33 |  | 61.45 | ± | 7.22 | 57.57 | ± | 6.46 | 36.91 | ± | 4.05 | 36.26 | ± | 5.44 |  |  |
| 17 | Ethyl benzoate | 1172 | 0.72 | ± | 0.14 | 0.91 | ± | 0.21 | 1.31 | ± | 0.48 | 0.88 | ± | 0.09 |  | 3.12 | ± | 0.14 | 3.80 | ± | 0.70 | 2.09 | ± | 0.49 | 3.58 | ± | 4.54 |  |  |
| 18 | 2-(2-Butoxyethoxy)ethanol | 1192 | 6.68 | ± | 0.45 | 7.53 | ± | 0.47 | 9.75 | ± | 0.22 | 7.85 | ± | 1.20 |  | 17.9 | ± | 1.96 | 12.20 | ± | 4.25 | 9.71 | ± | 0.36 | 9.14 | ± | 1.66 |  |  |
| 19 | Ethyl octanoate | 1194 | 0.09 | ± | 0.04 | 0.11 | ± | 0.07 | 0.12 | ± | 0.03 | 0.08 | ± | 0.03 |  | 0.27 | ± | 0.02 | 0.50 | ± | 0.19 | 0.20 | ± | 0.03 | 0.17 | ± | 0.04 |  |  |
| 20 | Methyl salicylate | 1195 | 7.49 | ± | 1.23 | 5.34 | ± | 3.68 | 7.33 | ± | 1.56 | 6.35 | ± | 0.42 |  | 11.08 | ± | 4.07 | 5.72 | ± | 0.76 | 4.51 | ± | 0.24 | 5.13 | ± | 0.36 |  |  |
| 21 | Octyl acetate | 1211 | 1.51 | ± | 0.32 | 1.14 | ± | 0.20 | 1.78 | ± | 0.25 | 2.10 | ± | 0.33 |  | 4.05 | ± | 0.44 | 3.97 | ± | 0.86 | 2.32 | ± | 0.21 | 2.50 | ± | 0.25 |  |  |
| 22 | 2-Nonanol, acetate | 1236 | 0.71 | ± | 0.38 | 0.74 | ± | 0.22 | 1.17 | ± | 0.23 | 0.92 | ± | 0.24 |  | 2.58 | ± | 0.04 | 1.45 | ± | 0.01 | 1.32 | ± | 0.29 | 0.78 | ± | 0.08 |  |  |
| 23 | Isopentyl hexanoate | 1245 | 0.16 | ± | 0.02 | 0.11 | ± | 0.08 | 0.44 | ± | 0.08 | 0.31 | ± | 0.02 |  | 1.35 | ± | 0.25 | 0.70 | ± | 0.10 | 0.54 | ± | 0.06 | 0.31 | ± | 0.06 |  |  |
| 24 | Methyl 3-hydroxyoctanoate | 1253 | 2.27 | ± | 1.88 | 1.28 | ± | 1.41 | 1.62 | ± | 0.70 | 1.36 | ± | 0.41 |  | 18.67 | ± | 2.21 | 4.34 | ± | 1.09 | 6.31 | ± | 0.94 | 3.20 | ± | 0.45 |  |  |
| 25 | Triacetin | 1346 | 0.56 | ± | 0.14 | 0.47 | ± | 0.04 | 0.76 | ± | 0.15 | 0.55 | ± | 0.06 |  | 1.23 | ± | 0.14 | 0.90 | ± | 0.18 | 0.81 | ± | 0.07 | 0.68 | ± | 0.11 |  |  |
| 26 | 2-(2-Butoxyethoxy)ethyl acetate | 1366 | 8.00 | ± | 0.85 | 7.30 | ± | 0.48 | 12.07 | ± | 0.80 | 9.43 | ± | 0.13 |  | 13.36 | ± | 1.85 | 12.76 | ± | 0.66 | 9.97 | ± | 0.86 | 8.96 | ± | 0.56 |  |  |
| 27 | Octyl 2-methylbutyrate | 1442 | 1.25 | ± | 0.55 | 0.57 | ± | 0.50 | 1.42 | ± | 0.28 | 1.05 | ± | 0.22 |  | 5.90 | ± | 1.18 | 3.27 | ± | 0.6 | 2.92 | ± | 0.13 | 1.88 | ± | 0.37 |  |  |
| 28 | Acetic acid, cinnamyl ester | 1449 | 2.77 | ± | 0.98 | 1.85 | ± | 1.55 | 4.06 | ± | 0.69 | 2.89 | ± | 0.77 |  | 13.96 | ± | 1.73 | 6.35 | ± | 0.72 | 6.78 | ± | 0.75 | 5.52 | ± | 0.68 |  |  |
| 29 | Octyl hexanoate | 1575 | 3.66 | ± | 2.86 | 0.18 | ± | 0.03 | 2.6 | ± | 3.18 | 0.57 | ± | 0.11 |  | 22.12 | ± | 3.01 | 3.72 | ± | 1.09 | 9.69 | ± | 1.59 | 6.73 | ± | 1.02 |  |  |
|  | ***Sum of esters*** | | 92.91 | ± | 14.82 | 104.10 | ± | 1.62 | 142.84 | ± | 8.49 | 130.63 | ± | 12.93 |  | 251.76 | ± | 18.11 | 210.81 | ± | 22.22 | 159.41 | ± | 8.68 | 138.48 | ± | 7.95 |  |  |
| *Monoterpene* | |  |  |  |  |  |  |  |  |  |  |  |  |  |  |  |  |  |  |  |  |  |  |  |  |  |  |  | |
| 30 | β-Ocimene | 1049 | 0.20 | ± | 0.03 | 0.11 | ± | 0.02 | 0.25 | ± | 0.01 | 0.24 | ± | 0.03 |  | 0.31 | ± | 0.10 | 0.46 | ± | 0.03 | 0.28 | ± | 0.05 | 0.25 | ± | 0.01 |  |  |
|  | ***Sum of monoterpens*** | | 0.20 | ± | 0.03 | 0.11 | ± | 0.02 | 0.25 | ± | 0.01 | 0.24 | ± | 0.03 |  | 0.31 | ± | 0.10 | 0.46 | ± | 0.03 | 0.28 | ± | 0.05 | 0.25 | ± | 0.01 |  |  |
| Sesquiterpenes | |  |  |  |  |  |  |  |  |  |  |  |  |  |  |  |  |  |  |  |  |  |  |  |  |  |  |  |  |
| 31 | (Z)-β-Famesene | 1442 | 7.83 | ± | 2.21 | 3.56 | ± | 0.89 | 9.62 | ± | 2.96 | 7.87 | ± | 1.06 |  | 23.43 | ± | 3.24 | 15.45 | ± | 3.00 | 14.20 | ± | 1.85 | 12.62 | ± | 1.03 |  |  |
| 32 | (E)-β-Farnesene | 1459 | 2.29 | ± | 1.09 | 1.00 | ± | 0.67 | 2.43 | ± | 0.8 | 2.01 | ± | 0.34 |  | 6.21 | ± | 1.08 | 4.06 | ± | 0.65 | 3.77 | ± | 0.59 | 2.59 | ± | 0.44 |  |  |
| 33 | (Z,E)-α-Farnesene | 1479 | 3.72 | ± | 1.84 | 1.43 | ± | 0.37 | 4.17 | ± | 1.08 | 3.18 | ± | 0.59 |  | 10.67 | ± | 1.92 | 6.61 | ± | 0.82 | 6.87 | ± | 1.07 | 5.15 | ± | 0.25 |  |  |
|  | ***Sum of sesquiterpenes*** | | 13.85 | ± | 5.13 | 6.00 | ± | 1.91 | 16.22 | ± | 4.81 | 13.06 | ± | 1.98 |  | 40.31 | ± | 6.22 | 26.12 | ± | 4.47 | 24.84 | ± | 3.48 | 20.37 | ± | 1.70 |  |  |
| *Acids* | |  |  |  |  |  |  |  |  |  |  |  |  |  |  |  |  |  |  |  |  |  |  |  |  |  |  |  | |
| 34 | 2-Ethylhexanoic acid | 1125 | 0.67 | ± | 0.07 | 0.73 | ± | 0.10 | 1.03 | ± | 0.06 | 0.82 | ± | 0.02 |  | 1.19 | ± | 0.10 | 1.11 | ± | 0.08 | 0.80 | ± | 0.06 | 0.78 | ± | 0.06 |  |  |
| 35 | Nonanoic acid | 1276 | 10.38 | ± | 2.33 | 10.32 | ± | 1.25 | 13.3 | ± | 1.88 | 8.37 | ± | 1.10 |  | 13.09 | ± | 1.99 | 13.42 | ± | 2.85 | 8.81 | ± | 0.51 | 9.55 | ± | 0.04 |  |  |
| 36 | Hydrocinnamic acid | 1350 | 0.00 | ± | 0.00 | 0.00 | ± | 0.00 | 0.00 | ± | 0.00 | 0.00 | ± | 0.00 |  | 0.10 | ± | 0.02 | 0.05 | ± | 0.01 | 0.05 | ± | 0.01 | 0.05 | ± | 0.04 |  |  |
|  | ***Sum of acids*** | | 11.06 | ± | 2.36 | 11.05 | ± | 1.22 | 14.32 | ± | 1.94 | 9.18 | ± | 1.08 |  | 14.37 | ± | 1.95 | 14.57 | ± | 2.88 | 9.66 | ± | 0.55 | 10.38 | ± | 0.09 |  |  |
| *Lactone* | |  |  |  |  |  |  |  |  |  |  |  |  |  |  |  |  |  |  |  |  |  |  |  |  |  |  |  | |
| 37 | γ-Dodecalactone | 1472 | 14.30 | ± | 6.92 | 6.09 | ± | 0.85 | 17.36 | ± | 2.90 | 12.36 | ± | 2.60 |  | 41.95 | ± | 5.49 | 24.24 | ± | 4.30 | 22.67 | ± | 2.01 | 16.43 | ± | 4.92 |  |  |
|  | ***Sum of lactones*** | | 14.30 | ± | 6.92 | 6.09 | ± | 0.85 | 17.36 | ± | 2.90 | 12.36 | ± | 2.60 |  | 41.95 | ± | 5.49 | 24.24 | ± | 4.30 | 22.67 | ± | 2.01 | 16.43 | ± | 4.92 |  |  |
| *Ketone* | |  |  |  |  |  |  |  |  |  |  |  |  |  |  |  |  |  |  |  |  |  |  |  |  |  |  |  | |
| 38 | 2-Heptanone | 891 | 3.08 | ± | 1.67 | 2.72 | ± | 1.20 | 3.12 | ± | 0.75 | 2.09 | ± | 0.09 |  | 9.15 | ± | 1.02 | 4.64 | ± | 0.91 | 5.78 | ± | 0.29 | 1.96 | ± | 0.24 |  |  |
|  | ***Sum of ketones*** | | 3.08 | ± | 1.67 | 2.72 | ± | 1.20 | 3.12 | ± | 0.75 | 2.09 | ± | 0.09 |  | 9.15 | ± | 1.02 | 4.64 | ± | 0.91 | 5.78 | ± | 0.29 | 1.96 | ± | 0.24 |  |  |
| *Furans* | |  |  |  |  |  |  |  |  |  |  |  |  |  |  |  |  |  |  |  |  |  |  |  |  |  |  |  | |
| 39 | Mesifuran | 1067 | 37.72 | ± | 4.27 | 31.91 | ± | 6.98 | 69.44 | ± | 7.52 | 52.38 | ± | 4.58 |  | 165.8 | ± | 1.51 | 110.99 | ± | 8.10 | 96.82 | ± | 9.96 | 91.18 | ± | 6.15 |  |  |
| 40 | Furaneol | 1081 | 0.02 | ± | 0.01 | 0.40 | ± | 0.05 | 4.41 | ± | 1.29 | 2.39 | ± | 1.11 |  | 5.27 | ± | 1.63 | 1.63 | ± | 0.68 | 2.05 | ± | 0.76 | 11.73 | ± | 4.94 |  |  |
|  | ***Sum of furans*** | | 37.74 | ± | 4.27 | 32.31 | ± | 7.02 | 73.85 | ± | 8.20 | 54.77 | ± | 3.74 |  | 171.07 | ± | 0.24 | 112.62 | ± | 7.56 | 98.88 | ± | 9.81 | 102.92 | ± | 11.09 |  |  |
| *Monoterpene alcohols* | |  |  |  |  |  |  |  |  |  |  |  |  |  |  |  |  |  |  |  |  |  |  |  |  |  |  |  | |
| 41 | (E)-Linalool oxide | 1086 | 2.05 | ± | 0.50 | 3.07 | ± | 1.62 | 4.19 | ± | 0.43 | 3.58 | ± | 0.77 |  | 6.45 | ± | 0.60 | 6.10 | ± | 1.09 | 5.39 | ± | 0.53 | 2.89 | ± | 0.85 |  |  |
| 42 | Linalool | 1099 | 55.81 | ± | 7.06 | 50.32 | ± | 6.77 | 79.13 | ± | 2.45 | 76.98 | ± | 2.64 |  | 118.61 | ± | 5.45 | 95.32 | ± | 2.91 | 97.55 | ± | 6.75 | 54.88 | ± | 2.25 |  |  |
| 43 | L-α-Terpineol | 1198 | 4.21 | ± | 0.70 | 3.80 | ± | 0.91 | 6.60 | ± | 0.74 | 5.40 | ± | 0.16 |  | 11.13 | ± | 1.12 | 11.03 | ± | 2.40 | 8.99 | ± | 0.78 | 5.60 | ± | 0.29 |  |  |
| 44 | Nerol | 1248 | 0.62 | ± | 0.24 | 0.6 | ± | 0.29 | 1.17 | ± | 0.05 | 1.08 | ± | 0.06 |  | 0.91 | ± | 0.15 | 1.66 | ± | 0.03 | 1.39 | ± | 0.17 | 0.77 | ± | 0.10 |  |  |
|  | ***Sum of monoterpene alcohols*** | | 62.69 | ± | 8.30 | 57.79 | ± | 9.26 | 91.09 | ± | 1.68 | 87.04 | ± | 3.04 |  | 137.10 | ± | 6.15 | 114.11 | ± | 2.63 | 113.33 | ± | 7.66 | 64.15 | ± | 3.19 |  |  |
| Sesquiterpene alcohols | |  |  |  |  |  |  |  |  |  |  |  |  |  |  |  |  |  |  |  |  |  |  |  |  |  |  |  |  |
| 45 | Nerolidol | 1558 | 129.15 | ± | 9.19 | 82.44 | ± | 6.26 | 165.63 | ± | 26.95 | 144.74 | ± | 18.57 |  | 338.97 | ± | 13.47 | 220.74 | ± | 21.12 | 155.43 | ± | 15.83 | 176.06 | ± | 15.84 |  |  |
| 46 | Bisabolol oxide II | 1660 | 2.99 | ± | 0.42 | 2.48 | ± | 0.84 | 4.98 | ± | 0.56 | 2.79 | ± | 0.40 |  | 6.42 | ± | 0.65 | 5.90 | ± | 0.63 | 4.98 | ± | 0.96 | 3.46 | ± | 0.69 |  |  |
| 47 | L-α-Bisabolol | 1688 | 0.00 | ± | 0.00 | 0.00 | ± | 0.00 | 0.00 | ± | 0.00 | 0.00 | ± | 0.00 |  | 0.00 | ± | 0.00 | 0.00 | ± | 0.00 | 0.00 | ± | 0.00 | 0.00 | ± | 0.00 |  |  |
|  | ***Sum of sesquiterpene alcohols*** | | 132.14 | ± | 9.61 | 84.92 | ± | 6.93 | 170.61 | ± | 27.46 | 147.53 | ± | 18.74 |  | 345.38 | ± | 14.12 | 226.65 | ± | 21.74 | 160.41 | ± | 16.78 | 179.52 | ± | 16.27 |  |  |
|  | ***Total*** | | 571.80 | ± | 45.23 | 567.14 | ± | 37.44 | 727.85 | ± | 29.93 | 615.91 | ± | 35.52 |  | 1263.08 | ± | 47.06 | 919.71 | ± | 44.45 | 775.55 | ± | 49.70 | 627.58 | ± | 41.72 |  |  |

(Continues…)

| **Compound name** | | | **RT** | **Storage time (days)** | | | | | | | | | | | | | | | | | | | | | | | | | |  |
| --- | --- | --- | --- | --- | --- | --- | --- | --- | --- | --- | --- | --- | --- | --- | --- | --- | --- | --- | --- | --- | --- | --- | --- | --- | --- | --- | --- | --- | --- | --- |
|  |  |  |  | **4 d** | | | | | | | | | | | |  | **5 d** | | | | | | | | | | | | |  |
|  |  |  |  | **10.6%** | | | **21.2%** | | | **35.0%** | | | **54.4%** | | |  | **10.6%** | | | **21.2%** | | | **35.0%** | | | **54.4%** | | |  |  |
| *Aldehydes* | | |  |  |  |  |  |  |  |  |  |  |  |  |  |  |  |  |  |  |  |  |  |  |  |  |  |  |  |  |
| 1 | | Hexanal | 804 | 11.04 | ± | 2.93 | 10.34 | ± | 1.29 | 8.22 | ± | 0.96 | 13.06 | ± | 3.68 |  | 11.58 | ± | 1.16 | 12.58 | ± | 0.36 | 55.06 | ± | 2.74 | 34.69 | ± | 6.74 |  |  |
| 2 | | (E)-2-Hexenal | 854 | 118.67 | ± | 10.45 | 100.91 | ± | 6.69 | 91.64 | ± | 5.87 | 160.01 | ± | 10.62 |  | 100.54 | ± | 4.34 | 126.06 | ± | 5.04 | 87.37 | ± | 9.34 | 84.04 | ± | 10.65 |  |  |
| 3 | | Heptanal | 901 | 0.30 | ± | 0.14 | 0.12 | ± | 0.02 | 0.17 | ± | 0.02 | 0.16 | ± | 0.01 |  | 0.24 | ± | 0.01 | 0.36 | ± | 0.07 | 0.39 | ± | 0.03 | 0.46 | ± | 0.04 |  |  |
| 4 | | (E,E)-2,4-Hexadienal | 908 | 0.20 | ± | 0.03 | 0.08 | ± | 0.02 | 0.08 | ± | 0.01 | 0.18 | ± | 0.01 |  | 0.02 | ± | 0.00 | 0.04 | ± | 0.01 | 0.01 | ± | 0.00 | 0.02 | ± | 0.00 |  |  |
| 5 | | Benzaldehyde | 963 | 0.45 | ± | 0.12 | 0.31 | ± | 0.03 | 0.25 | ± | 0.02 | 0.53 | ± | 0.05 |  | 0.26 | ± | 0.06 | 0.26 | ± | 0.03 | 0.75 | ± | 0.01 | 0.31 | ± | 0.03 |  |  |
| 6 | | Octanal | 1005 | 9.14 | ± | 6.96 | 13.71 | ± | 0.92 | 0.49 | ± | 0.01 | 0.47 | ± | 0.06 |  | 0.30 | ± | 0.19 | 0.35 | ± | 0.14 | 2.78 | ± | 0.21 | 1.98 | ± | 0.36 |  |  |
| 7 | | Decanal | 1205 | 1.10 | ± | 0.33 | 1.03 | ± | 0.13 | 1.95 | ± | 0.19 | 1.21 | ± | 0.23 |  | 1.16 | ± | 0.10 | 1.65 | ± | 0.08 | 1.42 | ± | 0.15 | 1.22 | ± | 0.14 |  |  |
|  | | ***Sum of aldehydes*** | | 140.89 | ± | 16.04 | 126.51 | ± | 7.45 | 102.79 | ± | 6.90 | 175.62 | ± | 14.34 |  | 114.12 | ± | 3.21 | 141.31 | ± | 5.60 | 147.79 | ± | 10.65 | 122.71 | ± | 17.51 |  |  |
| *Esters* | | |  |  |  |  |  |  |  |  |  |  |  |  |  |  |  |  |  |  |  |  |  |  |  |  |  |  |  | |
| 8 | | Methyl butanoate | 723 | 14.81 | ± | 1.56 | 16.35 | ± | 1.65 | 11.5 | ± | 1.34 | 23.12 | ± | 1.10 |  | 16.70 | ± | 1.63 | 25.91 | ± | 1.34 | 24.56 | ± | 7.64 | 30.12 | ± | 0.28 |  |  |
| 9 | | Methyl 2-methylbutyrate | 785 | 0.91 | ± | 0.22 | 0.79 | ± | 0.07 | 0.7 | ± | 0.03 | 1.28 | ± | 0.20 |  | 4.90 | ± | 0.25 | 2.82 | ± | 0.18 | 1.55 | ± | 0.48 | 2.34 | ± | 0.39 |  |  |
| 10 | | Isopropyl butyrate | 846 | 0.32 | ± | 0.06 | 0.28 | ± | 0.02 | 0.18 | ± | 0.02 | 0.48 | ± | 0.09 |  | 0.32 | ± | 0.02 | 0.49 | ± | 0.03 | 0.46 | ± | 0.01 | 0.47 | ± | 0.02 |  |  |
| 11 | | Methyl hexanoate | 926 | 19.62 | ± | 1.08 | 21.62 | ± | 1.57 | 13.67 | ± | 1.17 | 23.6 | ± | 0.90 |  | 24.14 | ± | 0.99 | 27.23 | ± | 3.17 | 33.82 | ± | 1.81 | 36.5 | ± | 0.65 |  |  |
| 12 | | Ethyl hexanoate | 998 | 3.31 | ± | 0.27 | 3.95 | ± | 0.69 | 17.91 | ± | 2.05 | 6.77 | ± | 0.75 |  | 8.18 | ± | 1.14 | 10.66 | ± | 3.65 | 132.91 | ± | 2.74 | 54.09 | ± | 3.03 |  |  |
| 13 | | Hexyl acetate | 1011 | 4.17 | ± | 1.10 | 2.61 | ± | 0.78 | 13.12 | ± | 0.68 | 5.12 | ± | 0.36 |  | 34.2 | ± | 1.50 | 59.77 | ± | 3.76 | 57.87 | ± | 4.97 | 5.65 | ± | 0.38 |  |  |
| 14 | | 2-Hexen-1-yl acetate | 1019 | 12.2 | ± | 2.09 | 5.9 | ± | 0.98 | 18.52 | ± | 3.11 | 19.96 | ± | 0.91 |  | 19.31 | ± | 3.10 | 32.27 | ± | 0.52 | 28.34 | ± | 0.79 | 2.78 | ± | 0.48 |  |  |
| 15 | | Methyl octanoate | 1026 | 1.51 | ± | 0.25 | 1.98 | ± | 0.84 | 1.31 | ± | 0.11 | 1.48 | ± | 0.04 |  | 3.95 | ± | 0.47 | 3.35 | ± | 0.15 | 4.89 | ± | 0.80 | 4.64 | ± | 0.44 |  |  |
| 16 | | Benzyl acetate | 1164 | 45.79 | ± | 1.83 | 37.39 | ± | 3.36 | 46.77 | ± | 2.29 | 72.09 | ± | 2.62 |  | 36.99 | ± | 0.55 | 54.77 | ± | 3.54 | 32.43 | ± | 3.01 | 31.59 | ± | 2.37 |  |  |
| 17 | | Ethyl benzoate | 1172 | 1.15 | ± | 0.06 | 1.59 | ± | 0.15 | 1.26 | ± | 0.08 | 1.71 | ± | 0.08 |  | 10.11 | ± | 0.62 | 2.28 | ± | 3.09 | 12.84 | ± | 0.89 | 9.75 | ± | 0.70 |  |  |
| 18 | | 2-(2-Butoxyethoxy)ethanol | 1192 | 14.58 | ± | 2.25 | 14.91 | ± | 0.92 | 16.59 | ± | 0.7 | 14.34 | ± | 1.96 |  | 10.79 | ± | 0.78 | 24.26 | ± | 2.85 | 34.81 | ± | 2.65 | 14.97 | ± | 1.78 |  |  |
| 19 | | Ethyl octanoate | 1194 | 0.24 | ± | 0.01 | 0.20 | ± | 0.00 | 2.51 | ± | 0.69 | 0.26 | ± | 0.05 |  | 3.71 | ± | 0.45 | 4.39 | ± | 0.64 | 33.06 | ± | 3.49 | 17.9 | ± | 6.28 |  |  |
| 20 | | Methyl salicylate | 1195 | 8.49 | ± | 0.52 | 3.27 | ± | 0.28 | 2.71 | ± | 0.44 | 8.21 | ± | 0.77 |  | 8.76 | ± | 0.38 | 12.09 | ± | 0.17 | 11.52 | ± | 0.44 | 11.04 | ± | 0.74 |  |  |
| 21 | | Octyl acetate | 1211 | 3.36 | ± | 0.47 | 1.88 | ± | 0.33 | 2.62 | ± | 0.11 | 3.31 | ± | 0.16 |  | 2.44 | ± | 0.09 | 4.06 | ± | 0.98 | 11.43 | ± | 0.69 | 3.39 | ± | 0.26 |  |  |
| 22 | | 2-Nonanol, acetate | 1236 | 1.48 | ± | 0.19 | 1.49 | ± | 0.07 | 1.51 | ± | 0.11 | 1.72 | ± | 0.09 |  | 2.14 | ± | 0.14 | 2.07 | ± | 0.26 | 2.27 | ± | 0.32 | 2.59 | ± | 0.11 |  |  |
| 23 | | Isopentyl hexanoate | 1245 | 1.17 | ± | 0.24 | 1.39 | ± | 0.14 | 0.84 | ± | 0.09 | 1.10 | ± | 0.11 |  | 2.31 | ± | 0.36 | 1.71 | ± | 0.12 | 4.88 | ± | 0.48 | 2.56 | ± | 0.18 |  |  |
| 24 | | Methyl 3-hydroxyoctanoate | 1253 | 15.51 | ± | 4.14 | 27.19 | ± | 1.01 | 12.20 | ± | 0.54 | 7.54 | ± | 0.88 |  | 36.45 | ± | 7.94 | 33.07 | ± | 1.71 | 33.24 | ± | 24.21 | 29.00 | ± | 0.76 |  |  |
| 25 | | Triacetin | 1346 | 0.87 | ± | 0.15 | 0.94 | ± | 0.09 | 0.88 | ± | 0.03 | 1.05 | ± | 0.15 |  | 1.25 | ± | 0.05 | 1.71 | ± | 0.07 | 1.76 | ± | 0.03 | 1.86 | ± | 0.10 |  |  |
| 26 | | 2-(2-Butoxyethoxy)ethyl acetate | 1366 | 9.86 | ± | 0.99 | 12.30 | ± | 1.73 | 10.00 | ± | 0.81 | 12.75 | ± | 0.31 |  | 10.23 | ± | 0.20 | 14.66 | ± | 0.82 | 12.94 | ± | 0.67 | 12.21 | ± | 1.06 |  |  |
| 27 | | Octyl 2-methylbutyrate | 1442 | 3.12 | ± | 0.74 | 3.49 | ± | 0.31 | 2.17 | ± | 0.07 | 2.93 | ± | 0.43 |  | 4.73 | ± | 0.01 | 5.02 | ± | 0.58 | 6.53 | ± | 0.36 | 6.33 | ± | 1.04 |  |  |
| 28 | | Acetic acid, cinnamyl ester | 1449 | 8.71 | ± | 2.13 | 5.22 | ± | 0.75 | 6.81 | ± | 1.64 | 7.44 | ± | 1.08 |  | 9.80 | ± | 0.75 | 12.92 | ± | 1.66 | 19.32 | ± | 1.82 | 11.47 | ± | 1.16 |  |  |
| 29 | | Octyl hexanoate | 1575 | 10.58 | ± | 6.78 | 16.96 | ± | 1.75 | 9.36 | ± | 0.98 | 8.52 | ± | 0.54 |  | 51.36 | ± | 2.33 | 25.18 | ± | 1.50 | 61.11 | ± | 4.93 | 35.37 | ± | 5.28 |  |  |
|  | | ***Sum of esters*** | | 181.78 | ± | 17.23 | 181.70 | ± | 8.20 | 193.15 | ± | 5.12 | 224.77 | ± | 6.04 |  | 302.78 | ± | 10.12 | 360.69 | ± | 0.59 | 562.56 | ± | 20.19 | 326.62 | ± | 15.59 |  |  |
| *Monoterpens* | | |  |  |  |  |  |  |  |  |  |  |  |  |  |  |  |  |  |  |  |  |  |  |  |  |  |  |  | |
| 30 | | β-Ocimene | 1049 | 0.31 | ± | 0.08 | 0.47 | ± | 0.06 | 0.26 | ± | 0.02 | 0.47 | ± | 0.08 |  | 0.28 | ± | 0.03 | 0.44 | ± | 0.08 | 0.32 | ± | 0.03 | 0.37 | ± | 0.03 |  |  |
|  | | ***Sum of monoterpenes*** | | 0.31 | ± | 0.08 | 0.47 | ± | 0.06 | 0.26 | ± | 0.02 | 0.47 | ± | 0.08 |  | 0.28 | ± | 0.03 | 0.44 | ± | 0.08 | 0.32 | ± | 0.03 | 0.37 | ± | 0.03 |  |  |
| Sesquiterpenes | | |  |  |  |  |  |  |  |  |  |  |  |  |  |  |  |  |  |  |  |  |  |  |  |  |  |  |  |  |
| 31 | | (Z)-β-Famesene | 1442 | 10.91 | ± | 1.98 | 15.34 | ± | 1.71 | 13.26 | ± | 1.24 | 19.18 | ± | 1.21 |  | 15.73 | ± | 0.86 | 19.67 | ± | 2.15 | 25.37 | ± | 0.79 | 20.76 | ± | 0.34 |  |  |
| 32 | | (E)-β-Farnesene | 1459 | 2.57 | ± | 0.26 | 4.07 | ± | 0.39 | 3.12 | ± | 0.75 | 3.67 | ± | 0.42 |  | 4.08 | ± | 0.98 | 4.55 | ± | 0.23 | 6.59 | ± | 0.05 | 5.02 | ± | 0.30 |  |  |
| 33 | | (Z,E)-α-Farnesene | 1479 | 4.52 | ± | 0.51 | 7.17 | ± | 0.85 | 5.29 | ± | 1.37 | 6.29 | ± | 2.13 |  | 6.32 | ± | 1.00 | 8.04 | ± | 0.70 | 11.69 | ± | 0.18 | 8.95 | ± | 0.30 |  |  |
|  | | ***Sum of sesquiterpenes*** | | 18.00 | ± | 2.73 | 26.59 | ± | 2.41 | 21.68 | ± | 3.36 | 29.14 | ± | 3.43 |  | 26.13 | ± | 2.79 | 32.27 | ± | 3.06 | 43.65 | ± | 1.01 | 34.73 | ± | 0.76 |  |  |
| *Acids* | | |  |  |  |  |  |  |  |  |  |  |  |  |  |  |  |  |  |  |  |  |  |  |  |  |  |  |  | |
| 34 | | 2-Ethylhexanoic acid | 1125 | 0.93 | ± | 0.03 | 1.15 | ± | 0.18 | 1.00 | ± | 0.07 | 1.06 | ± | 0.14 |  | 1.01 | ± | 0.06 | 1.48 | ± | 0.14 | 1.22 | ± | 0.08 | 1.27 | ± | 0.11 |  |  |
| 35 | | Nonanoic acid | 1276 | 9.96 | ± | 0.98 | 18.97 | ± | 2.71 | 16.83 | ± | 1.22 | 9.75 | ± | 1.48 |  | 10.31 | ± | 0.48 | 14.6 | ± | 0.25 | 10.01 | ± | 0.58 | 12.28 | ± | 0.87 |  |  |
| 36 | | Hydrocinnamic acid | 1350 | 0.07 | ± | 0.02 | 0.10 | ± | 0.00 | 0.06 | ± | 0.01 | 0.07 | ± | 0.01 |  | 0.13 | ± | 0.01 | 0.11 | ± | 0.02 | 0.16 | ± | 0.01 | 0.37 | ± | 0.26 |  |  |
|  | | ***Sum of acids*** | | 10.97 | ± | 0.95 | 20.21 | ± | 2.87 | 17.88 | ± | 1.28 | 10.88 | ± | 1.47 |  | 11.45 | ± | 0.52 | 16.19 | ± | 0.40 | 11.39 | ± | 0.57 | 13.91 | ± | 0.92 |  |  |
| *Lactone* | | |  |  |  |  |  |  |  |  |  |  |  |  |  |  |  |  |  |  |  |  |  |  |  |  |  |  |  | |
| 37 | | γ-Dodecalactone | 1472 | 16.35 | ± | 3.35 | 28.04 | ± | 2.24 | 12.66 | ± | 1.79 | 12.11 | ± | 0.80 |  | 17.53 | ± | 4.57 | 15.51 | ± | 0.82 | 46.11 | ± | 2.79 | 36.52 | ± | 0.57 |  |  |
|  | | ***Sum of lactones*** | | 16.35 | ± | 3.35 | 28.04 | ± | 2.24 | 12.66 | ± | 1.79 | 12.11 | ± | 0.80 |  | 17.53 | ± | 4.57 | 15.51 | ± | 0.82 | 46.11 | ± | 2.79 | 36.52 | ± | 0.57 |  |  |
| *Ketone* | | |  |  |  |  |  |  |  |  |  |  |  |  |  |  |  |  |  |  |  |  |  |  |  |  |  |  |  | |
| 38 | | 2-Heptanone | 891 | 5.09 | ± | 1.11 | 4.51 | ± | 0.26 | 3.13 | ± | 0.32 | 5.12 | ± | 0.09 |  | 4.28 | ± | 0.27 | 8.41 | ± | 0.42 | 7.67 | ± | 0.84 | 7.59 | ± | 0.46 |  |  |
|  | ***Sum of ketones*** | | | 5.09 | ± | 1.11 | 4.51 | ± | 0.26 | 3.13 | ± | 0.32 | 5.12 | ± | 0.09 |  | 4.28 | ± | 0.27 | 8.41 | ± | 0.42 | 7.67 | ± | 0.84 | 7.59 | ± | 0.46 |  | |
| *Furans* | | |  |  |  |  |  |  |  |  |  |  |  |  |  |  |  |  |  |  |  |  |  |  |  |  |  |  |  | |
| 39 | | Mesifuran | 1067 | 142.2 | ± | 17.47 | 145.7 | ± | 4.23 | 142.85 | ± | 1.68 | 187.9 | ± | 17.84 |  | 179.87 | ± | 9.12 | 246.91 | ± | 18.31 | 203.54 | ± | 13.93 | 205.18 | ± | 11.46 |  |  |
| 40 | | Furaneol | 1081 | 0.20 | ± | 0.02 | 5.21 | ± | 0.20 | 19.30 | ± | 4.37 | 10.41 | ± | 2.39 |  | 46.03 | ± | 6.08 | 50.59 | ± | 4.84 | 36.26 | ± | 4.41 | 44.16 | ± | 9.66 |  |  |
|  | | ***Sum of furans*** | | 142.40 | ± | 17.49 | 150.91 | ± | 4.35 | 162.16 | ± | 5.79 | 198.31 | ± | 19.51 |  | 225.90 | ± | 10.58 | 297.49 | ± | 20.19 | 239.80 | ± | 17.00 | 249.33 | ± | 21.09 |  |  |
| *Monoterpene alcohols* | | |  |  |  |  |  |  |  |  |  |  |  |  |  |  |  |  |  |  |  |  |  |  |  |  |  |  |  | |
| 41 | | (E)-Linalool oxide | 1086 | 5.51 | ± | 1.25 | 4.73 | ± | 0.33 | 4.09 | ± | 0.39 | 5.78 | ± | 0.07 |  | 1.76 | ± | 0.04 | 7.83 | ± | 0.53 | 8.78 | ± | 0.20 | 1.39 | ± | 0.03 |  |  |
| 42 | | Linalool | 1099 | 73.65 | ± | 3.07 | 92.72 | ± | 2.95 | 66.17 | ± | 5.64 | 116.47 | ± | 1.64 |  | 65.33 | ± | 4.59 | 93.98 | ± | 8.33 | 76.09 | ± | 6.68 | 75.81 | ± | 1.06 |  |  |
| 43 | | L-α-Terpineol | 1198 | 13.44 | ± | 0.24 | 16.64 | ± | 1.89 | 7.56 | ± | 1.13 | 18.8 | ± | 2.33 |  | 10.37 | ± | 0.9 | 17 | ± | 2.49 | 9.94 | ± | 1.3 | 11.16 | ± | 0.21 |  |  |
| 44 | | Nerol | 1248 | 1.72 | ± | 0.59 | 2.30 | ± | 0.87 | 1.36 | ± | 0.36 | 2.79 | ± | 0.31 |  | 1.31 | ± | 0.1 | 1.62 | ± | 0.19 | 1.67 | ± | 0.12 | 1.46 | ± | 0.15 |  |  |
|  | | ***Sum of monoterpene alcohols*** | | 94.33 | ± | 2.62 | 116.40 | ± | 1.86 | 79.18 | ± | 7.31 | 143.84 | ± | 1.97 |  | 78.76 | ± | 3.79 | 120.43 | ± | 10.11 | 96.47 | ± | 6.98 | 89.81 | ± | 0.85 |  |  |
| Sesquiterpene alcohols | | |  |  |  |  |  |  |  |  |  |  |  |  |  |  |  |  |  |  |  |  |  |  |  |  |  |  |  |  |
| 45 | | Nerolidol | 1558 | 153.12 | ± | 20.05 | 193.88 | ± | 9.68 | 165.44 | ± | 14.89 | 215.01 | ± | 15.75 |  | 187.53 | ± | 2.68 | 224.17 | ± | 12.9 | 285.31 | ± | 8.09 | 233.42 | ± | 15.47 |  |  |
| 46 | | Bisabolol oxide II | 1660 | 4.72 | ± | 0.45 | 6.32 | ± | 0.63 | 5.01 | ± | 1.15 | 6.25 | ± | 0.83 |  | 5.35 | ± | 0.5 | 7.38 | ± | 0.5 | 6.91 | ± | 0.35 | 5.7 | ± | 0.52 |  |  |
| 47 | | L-α-Bisabolol | 1688 | 0.00 | ± | 0.00 | 0.00 | ± | 0.00 | 0.00 | ± | 0.00 | 0.00 | ± | 0.00 |  | 0.00 | ± | 0.00 | 0.00 | ± | 0.00 | 0.00 | ± | 0.00 | 0.00 | ± | 0.00 |  |  |
|  | | ***Sum of sesquiterpene alcohols*** | | 157.84 | ± | 20.49 | 200.20 | ± | 9.61 | 170.46 | ± | 15.89 | 221.25 | ± | 16.28 |  | 192.88 | ± | 2.97 | 231.55 | ± | 13.39 | 292.22 | ± | 8.10 | 239.12 | ± | 15.00 |  |  |
|  | | ***Totals*** | | 785.71 | ± | 76.49 | 882.95 | ± | 28.45 | 786.57 | ± | 34.82 | 1049.77 | ± | 52.86 |  | 996.78 | ± | 34.24 | 1252.58 | ± | 47.48 | 1474.82 | ± | 52.92 | 1148.06 | ± | 49.92 |  |  |

(Continues…)

| **Compound name** | | **RT** | **Storage time (days)** | | | | | | | | | | | |
| --- | --- | --- | --- | --- | --- | --- | --- | --- | --- | --- | --- | --- | --- | --- |
|  |  |  | **6 d** | | | | | | | | | | | |
|  |  |  | **10.6%** | | | **21.2%** | | | **35.0%** | | | **54.4%** | | |
| *Aldehydes* | |  |  |  |  |  |  |  |  |  |  |  |  |  |
| 1 | Hexanal | 804 | 91.49 | ± | 10.30 | 125.96 | ± | 7.01 | 114.69 | ± | 6.361 | 97.46 | ± | 9.52 |
| 2 | (E)-2-Hexenal | 854 | 98.90 | ± | 7.46 | 71.19 | ± | 7.72 | 103.24 | ± | 6.59 | 127.44 | ± | 3.43 |
| 3 | Heptanal | 901 | 0.71 | ± | 0.06 | 0.90 | ± | 0.03 | 0.54 | ± | 0.04 | 0.71 | ± | 0.05 |
| 4 | (E,E)-2,4-Hexadienal | 908 | 0.04 | ± | 0.00 | 0.01 | ± | 0.00 | 0.04 | ± | 0.01 | 0.03 | ± | 0.00 |
| 5 | Benzaldehyde | 963 | 0.86 | ± | 0.14 | 0.64 | ± | 0.04 | 1.30 | ± | 0.13 | 1.27 | ± | 0.15 |
| 6 | Octanal | 1005 | 7.05 | ± | 0.35 | 2.98 | ± | 0.05 | 1.84 | ± | 0.13 | 1.57 | ± | 0.12 |
| 7 | Decanal | 1205 | 7.42 | ± | 1.81 | 0.69 | ± | 0.07 | 2.05 | ± | 0.41 | 0.39 | ± | 0.01 |
|  | ***Sum of aldehydes*** | | 206.47 | ± | 17.82 | 191.11 | ± | 13.49 | 276.63 | ± | 16.77 | 228.87 | ± | 8.99 |
| *Esters* | |  |  |  |  |  |  |  |  |  |  |  |  |  |
| 8 | Methyl butanoate | 723 | 23.00 | ± | 1.15 | 12.03 | ± | 2.00 | 13.45 | ± | 0.49 | 16.97 | ± | 1.21 |
| 9 | Methyl 2-methylbutyrate | 785 | 7.75 | ± | 0.22 | 4.97 | ± | 3.16 | 3.43 | ± | 0.43 | 5.07 | ± | 0.23 |
| 10 | Isopropyl butyrate | 846 | 0.64 | ± | 0.09 | 0.1 | ± | 0.02 | 0.12 | ± | 0.01 | 0.25 | ± | 0.04 |
| 11 | Methyl hexanoate | 926 | 47.58 | ± | 1.53 | 45.61 | ± | 5.72 | 44.92 | ± | 4.62 | 37.4 | ± | 0.90 |
| 12 | Ethyl hexanoate | 998 | 271.11 | ± | 12.69 | 357.66 | ± | 8.07 | 538.52 | ± | 14.21 | 270.42 | ± | 7.26 |
| 13 | Hexyl acetate | 1011 | 6.36 | ± | 0.39 | 18.49 | ± | 3.94 | 76.76 | ± | 9.97 | 15.44 | ± | 0.47 |
| 14 | 2-Hexen-1-yl acetate | 1019 | 2.90 | ± | 0.52 | 2.69 | ± | 0.01 | 6.44 | ± | 1.16 | 22.33 | ± | 1.70 |
| 15 | Methyl octanoate | 1026 | 7.04 | ± | 1.04 | 9.46 | ± | 0.88 | 5.85 | ± | 3.03 | 7.01 | ± | 0.33 |
| 16 | Benzyl acetate | 1164 | 21.22 | ± | 5.02 | 19.99 | ± | 0.47 | 31.14 | ± | 6.64 | 23.37 | ± | 1.46 |
| 17 | Ethyl benzoate | 1172 | 14.37 | ± | 1.18 | 22.14 | ± | 1.78 | 28.23 | ± | 4.19 | 21.81 | ± | 0.85 |
| 18 | 2-(2-Butoxyethoxy)ethanol | 1192 | 10.95 | ± | 1.28 | 14.7 | ± | 0.23 | 19.09 | ± | 6.80 | 7.40 | ± | 0.29 |
| 19 | Ethyl octanoate | 1194 | 49.3 | ± | 2.26 | 76.84 | ± | 2.84 | 84.5 | ± | 16.07 | 67.39 | ± | 5.07 |
| 20 | Methyl salicylate | 1195 | 20.28 | ± | 1.78 | 4.52 | ± | 0.40 | 8.44 | ± | 2.02 | 3.86 | ± | 0.14 |
| 21 | Octyl acetate | 1211 | 12.82 | ± | 0.84 | 57.11 | ± | 6.42 | 52.33 | ± | 3.99 | 19.99 | ± | 2.46 |
| 22 | 2-Nonanol, acetate | 1236 | 2.44 | ± | 0.38 | 5.20 | ± | 0.70 | 3.71 | ± | 0.17 | 2.90 | ± | 0.40 |
| 23 | Isopentyl hexanoate | 1245 | 7.52 | ± | 1.11 | 4.73 | ± | 0.36 | 3.30 | ± | 0.23 | 2.14 | ± | 0.13 |
| 24 | Methyl 3-hydroxyoctanoate | 1253 | 78.95 | ± | 8.02 | 77.61 | ± | 3.54 | 49.77 | ± | 2.84 | 46.5 | ± | 2.46 |
| 25 | Triacetin | 1346 | 1.63 | ± | 0.17 | 1.84 | ± | 0.21 | 1.66 | ± | 0.14 | 1.71 | ± | 0.21 |
| 26 | 2-(2-Butoxyethoxy)ethyl acetate | 1366 | 12.34 | ± | 1.35 | 11.05 | ± | 0.25 | 13.78 | ± | 0.75 | 11.21 | ± | 0.50 |
| 27 | Octyl 2-methylbutyrate | 1442 | 6.07 | ± | 0.65 | 6.46 | ± | 1.81 | 7.12 | ± | 0.14 | 5.07 | ± | 0.71 |
| 28 | Acetic acid, cinnamyl ester | 1449 | 17.62 | ± | 2.62 | 28.46 | ± | 1.88 | 28.62 | ± | 5.30 | 15.38 | ± | 4.36 |
| 29 | Octyl hexanoate | 1575 | 100.44 | ± | 10.74 | 61.55 | ± | 1.77 | 65.59 | ± | 4.48 | 54.01 | ± | 2.14 |
|  | ***Sum of esters*** | | 722.33 | ± | 41.47 | 843.21 | ± | 17.80 | 1086.80 | ± | 12.56 | 657.65 | ± | 12.10 |
| *Monoterpene* | |  |  |  |  |  |  |  |  |  |  |  |  |  |
| 30 | β-Ocimene | 1049 | 0.09 | ± | 0.04 | 0.00 | ± | 0.00 | 0.00 | ± | 0.00 | 0.17 | ± | 0.03 |
|  | ***Sum of monoterpenes*** | | 0.09 | ± | 0.04 | 0.00 | ± | 0.00 | 0.00 | ± | 0.00 | 0.17 | ± | 0.03 |
| Sesquiterpenes | |  |  |  |  |  |  |  |  |  |  |  |  |  |
| 31 | (Z)-β-Famesene | 1442 | 17.16 | ± | 1.28 | 19.61 | ± | 2.58 | 28.7 | ± | 3.01 | 18.91 | ± | 1.09 |
| 32 | (E)-β-Farnesene | 1459 | 4.28 | ± | 0.36 | 5.19 | ± | 0.12 | 6.63 | ± | 0.86 | 4.8 | ± | 0.23 |
| 33 | (Z,E)-α-Farnesene | 1479 | 7.71 | ± | 0.86 | 8.94 | ± | 1.34 | 10.47 | ± | 0.83 | 8.55 | ± | 0.91 |
|  | ***Sum of sesquiterpenes*** |  | 29.15 | ± | 2.42 | 33.74 | ± | 3.75 | 45.80 | ± | 3.87 | 32.26 | ± | 1.78 |
| *Acids* | |  |  |  |  |  |  |  |  |  |  |  |  |  |
| 34 | 2-Ethylhexanoic acid | 1125 | 1.09 | ± | 0.14 | 1.22 | ± | 0.08 | 1.48 | ± | 0.26 | 1.38 | ± | 0.20 |
| 35 | Nonanoic acid | 1276 | 21.87 | ± | 2.07 | 9.20 | ± | 0.80 | 3.90 | ± | 2.88 | 21.58 | ± | 0.99 |
| 36 | Hydrocinnamic acid | 1350 | 0.30 | ± | 0.03 | 0.12 | ± | 0.03 | 0.11 | ± | 0.02 | 0.23 | ± | 0.03 |
|  | ***Sum of acids*** | | 23.36 | ± | 2.15 | 10.54 | ± | 0.83 | 5.50 | ± | 2.62 | 23.18 | ± | 0.82 |
| *Lactone* | |  |  |  |  |  |  |  |  |  |  |  |  |  |
| 37 | γ-Dodecalactone | 1472 | 30.98 | ± | 2.43 | 25.6 | ± | 3.31 | 40.28 | ± | 3.57 | 21.52 | ± | 1.81 |
|  | ***Sum of lactones*** | | 30.98 | ± | 2.43 | 25.6 | ± | 3.31 | 40.28 | ± | 3.57 | 21.52 | ± | 1.81 |
| *Ketone* | |  |  |  |  |  |  |  |  |  |  |  |  |  |
| 38 | 2-Heptanone | 891 | 12.80 | ± | 0.50 | 4.27 | ± | 0.76 | 6.53 | ± | 0.72 | 11.41 | ± | 1.34 |
|  | ***Sum of ketones*** |  | 12.80 | ± | 0.50 | 4.27 | ± | 0.76 | 6.53 | ± | 0.72 | 11.41 | ± | 1.34 |
| *Furans* | |  |  |  |  |  |  |  |  |  |  |  |  |  |
| 39 | Mesifuran | 1067 | 327.54 | ± | 16.92 | 305.44 | ± | 7.37 | 349.01 | ± | 11.92 | 281.1 | ± | 8.75 |
| 40 | Furaneol | 1081 | 69.31 | ± | 9.64 | 46.70 | ± | 9.24 | 57.09 | ± | 5.38 | 67.30 | ± | 2.46 |
|  |  |  | 396.86 | ± | 21.04 | 352.13 | ± | 3.27 | 406.10 | ± | 8.89 | 348.40 | ± | 10.13 |
| *Monoterpene alcohols* | |  |  |  |  |  |  |  |  |  |  |  |  |  |
| 41 | (E)-Linalool oxide | 1086 | 11.97 | ± | 0.77 | 11.57 | ± | 1.21 | 4.37 | ± | 0.13 | 10.97 | ± | 1.14 |
| 42 | Linalool | 1099 | 73.69 | ± | 6.10 | 78.09 | ± | 2.48 | 82.32 | ± | 2.00 | 83.26 | ± | 1.75 |
| 43 | L-α-Terpineol | 1198 | 11.12 | ± | 2.03 | 12.15 | ± | 0.76 | 10.89 | ± | 3.83 | 12.62 | ± | 0.47 |
| 44 | Nerol | 1248 | 1.99 | ± | 0.45 | 1.31 | ± | 0.15 | 0.85 | ± | 0.07 | 1.61 | ± | 0.14 |
|  | ***Sum of monoterpene alcohols*** | | 98.77 | ± | 8.38 | 103.12 | ± | 1.28 | 98.43 | ± | 4.13 | 108.46 | ± | 0.96 |
| Sesquiterpene alcohols | |  |  |  |  |  |  |  |  |  |  |  |  |  |
| 45 | Nerolidol | 1558 | 217.48 | ± | 15.14 | 247.61 | ± | 13.45 | 325.67 | ± | 38.43 | 234.3 | ± | 11.07 |
| 46 | Bisabolol oxide II | 1660 | 5.06 | ± | 3.90 | 5.85 | ± | 0.14 | 6.91 | ± | 0.48 | 6.69 | ± | 0.64 |
| 47 | L-α-Bisabolol | 1688 | 0.00 | ± | 0.00 | 0.00 | ± | 0.00 | 0.00 | ± | 0.00 | 0.00 | ± | 0.00 |
|  | ***Sum of sesquiterpene alcohols*** | | 222.54 | ± | 15.00 | 253.46 | ± | 13.33 | 332.58 | ± | 38.27 | 240.99 | ± | 11.61 |
|  | ***Totals*** | | 1766.93 | ± | 94.85 | 1843.92 | ± | 32.32 | 2337.65 | ± | 54.67 | 1700.83 | ± | 3.24 |

**Supplementary FIGURE 1.** Total phenolic
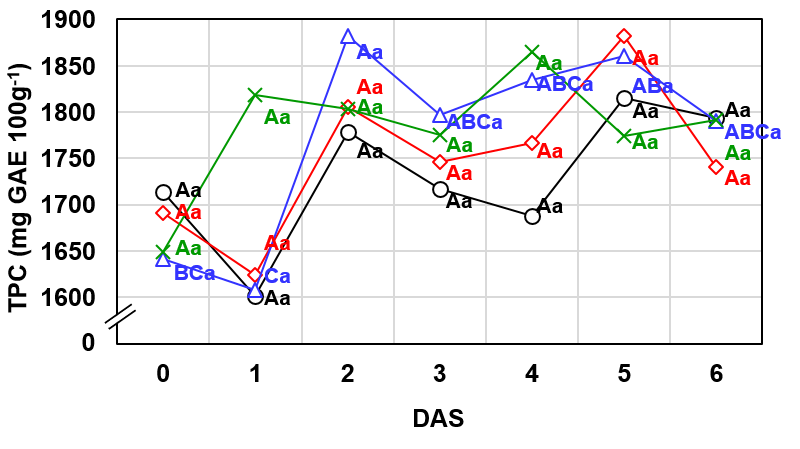
s content (TPC; mg GAE 100 g^-1^). Five concentrations (1.25-20 mM) of gallic acid were used to draw calibration curve. And result was calculated based on the calibration curve and expressed by gallic acid equivalent (GAE).

**
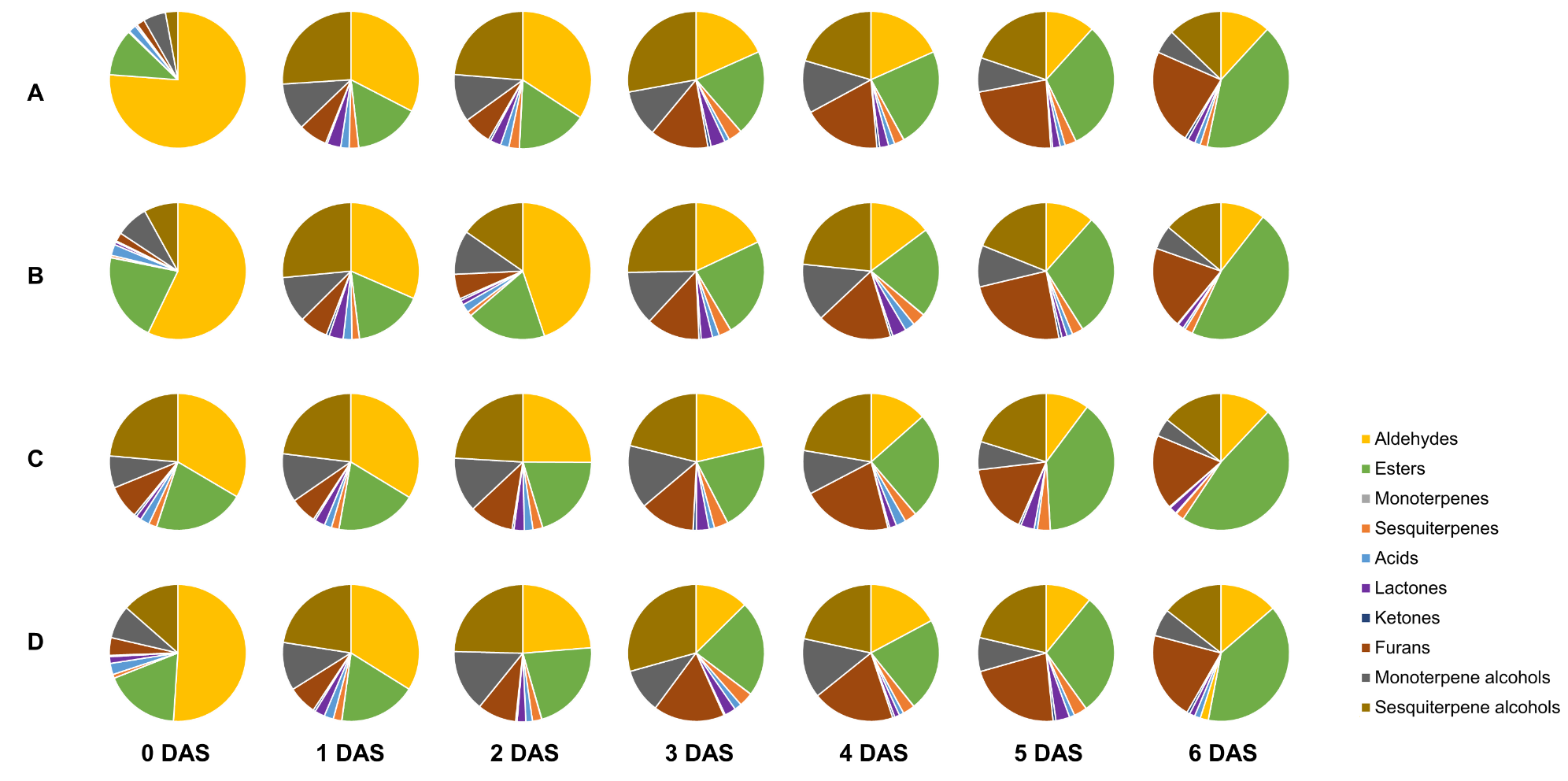
**

**Supplementary FIGURE 2.** Percentage change of VOCs groups by treatments including T1 (12.0%, **A**), T2 (25.3%, **B**), T3 (36.4%, **C**), and T4 (56.5%, **D**) according to days after storage (DAS).
